# A Fast-Forward Dilute-and-Shoot Multielement Method for Analysis of 33 Elements in Human Whole Blood, Serum, and Urine by Inductively Coupled Plasma Mass Spectrometry: A Streamlined Approach for Clinical Diagnostic and Biomonitoring

**DOI:** 10.1155/2024/9944995

**Published:** 2024-08-20

**Authors:** Sandra Huber, Jörg Michel, Maurice Reijnen, Maria Averina, Bjørn Bolann, Jon Øyvind Odland, Solrunn Hansen, Jan Brox

**Affiliations:** ^1^ Department of Laboratory Medicine University Hospital of North Norway, Tromsø NO-9038, Norway; ^2^ Perkin Elmer, Rodgau DE-63110, Germany; ^3^ Inorganic Solutions, Ravenstein NL-5371 CN, Netherlands; ^4^ Department of Clinical Medicine UiT The Arctic University of Norway, Tromsø NO-9037, Norway; ^5^ Department of Clinical Science University of Bergen, Bergen NO-5021, Norway; ^6^ Department of Medical Biochemistry and Pharmacology Haukeland University Hospital, Bergen NO-5021, Norway; ^7^ Bioscience and Aquaculture Nord University, Bodø NO-8049, Norway; ^8^ Department of Health and Care Sciences UiT The Arctic University of Norway, Tromsø NO-9037, Norway

## Abstract

The analysis of toxic and essential elements in human matrices is used in clinical diagnostics and for biomonitoring of different populations to study related health outcomes. This work aimed to develop fast and reliable methods for the analysis of a broad range of elements in liquid human matrices, such as whole blood, serum, and urine, with a similar setup for the three matrices and different analysis needs. An easy and fast-forward dilute-and-shoot method for 33 elements (i.e., Ag, Al, As, B, Ba, Be, Bi, Cd, Ce, Co, Cr, Cu, Hg, I, Li, Mn, Mo, Ni, Pb, Pd, Pt, Sb, Se, Sn, Sr, Te, Th, Tl, U, V, W, Zn, and Zr) was developed. 200 *µ*L of either sample material was diluted with an alkaline reagent to a volume of 4 mL in total. Sample dilution and preparation of matrix-matched calibration standards were performed in 48-well plates by an automated liquid handler. Diluted samples were analyzed by inductively coupled plasma mass spectrometry on a Perkin Elmer NexIon 300D ICP-MS instrument equipped with an ESI-FAST SC2DX autosampler in kinetic energy discrimination mode with helium as cell gas at either 4.8 mL or 5.7 mL and 1600 W RF generator power. The method validation results showed good accuracy for fresh human samples from an external quality assessment scheme with measured concentrations within the assigned concentration ranges. Good precision and reproducibility for most elements were demonstrated with variation coefficients below or far below 8% and 15% for whole blood, 8% and 10% for serum, and 10% and 10% for urine, respectively. The developed reagent and instrumental setup were applicable to all three matrices. This minimizes the risk of human errors when switching between analyses of the different sample matrices and allows a rapid and easy analysis of whole blood, serum, and urine within one day if needed. The method demonstrated robustness over time, withstanding minor changes in the preparation of working solutions and samples, instrumental analysis, and setup. Analysis of human real samples showed the method's applicability for 33 toxic and essential elements in whole blood, serum, and urine and at concentrations relevant to clinical diagnostics as well as biomonitoring.

## 1. Introduction

Both toxic and essential elements are ubiquitous in our everyday lives as they are omnipresent in the environment (e.g., air, water, and soil), food items, and consumer products. Essential elements, for example, copper (Cu), chromium (Cr), iodine (I), selenium (Se) or zinc (Zn), and cobalt (Co as a component in vitamin B12 [1]) and potential essential elements such as lithium (Li; [2]) play an important role in the human body. They are involved in many physiological processes [3], and their deficiency will lead to serious health problems in metabolic, neurodevelopmental, immunological, and reproductive functions [4]. However, when administrated in high doses, these elements can become toxic [5]. Toxic elements, for example, aluminum (Al), cadmium (Cd), lead (Pb), or mercury (Hg), pose potential risks to humans. They have no biological role in humans. Al is suspected of causing neurological disorders [6] and is associated with macrophagic myofasciitis as a result of its use in vaccines [7]. Hg is linked to movement disorders, neurodegeneration, and autoimmunity [8], while Pb affects early childhood development [9], especially in the central nervous system [10, 11]. Cd is associated with reduced kidney function [12], cardiovascular toxicity, and an increased risk for lung, kidney, prostate, and pancreatic cancers [13].

Analyses of toxic and essential elements in human matrices are widely used in clinical practice to diagnose intoxication and malnutrition. However, screening for such elements is also a valuable tool for biomonitoring the general population of different ages and genders to determine nutritional status, environmental exposure, and health effects [8, 9, 12, 14–26]. Occupational exposure monitoring of specific working groups exposed to elements such as Cd, Co, Cr, Hg, or Pb and others is strongly recommended, and in several countries, it is mandatory [24, 27–29]. For these purposes, robust methods are required to provide reliable and fast analysis of single or multiple elements consecutively. The importance of such population studies should not be underestimated, as the results are used by different professions, where the outcome is translated to health and food safety authorities as well as environmental agencies for decision-making and an update of national and local food intake recommendations.

Well-established methods for analyzing elements in human fluids are described in previously published literature studies, where different analytical tools, such as atomic absorption spectroscopy (AAS), inductively coupled plasma optical emission spectroscopy (ICP-OES), and inductively coupled plasma mass spectrometry (ICP-MS), were found to be adequate for analyzing elements in human fluids. The AAS technique is relatively simple, easy to implement, and cost-effective. However, some instrumental limitations, such as the number of elements analyzed consecutively, make the AAS rather inadequate for multielement analysis. In recent years, ICP-OES has become a common and widespread analysis technique used in various fields, including clinical diagnosis. It is cheaper in purchase, maintenance, and operation than ICP-MS. However, the detection limits and risk of spectral interferences are generally higher compared to the ICP-MS technology. ICP-MS is the instrument of choice when aiming for successive analysis of a broad range of elements with specific identification and low detection limits [22, 30–36] combined with a wide linear calibration range, short analysis time, and simple sample preparation [22, 32–35].

Before instrumental analysis, the samples need to be diluted or digested to guarantee a homogenous sample introduction to the ion source and minimise matrix interferences. In previously published literature, many different approaches are described. They range from a simple dilution with either acidic or alkaline reagents to rather time-consuming and more advanced acidic or alkaline digestion, which is sometimes combined with other oxidation reagents [30, 37]. The different approaches provide different advantages and disadvantages. Sample digestion followed by dilution provides very homogenous samples and is applied in several published studies [16, 21, 38–41]. However, this approach is time-consuming due to several sample preparation steps, the application of reusable equipment with the need for extra cleaning, and the potential background contamination from using higher volumes of digestion reagents.

Furthermore, a supplementary device for sample digestion, such as an advanced microwave device or a simple heating block, is needed. A simple dilution process is fast, and little background contamination is introduced due to few sample preparation steps. However, analysis can be challenging for matrices with a high content of salts and proteins with a higher risk of clogging the sample introduction parts on the instrument and matrix as well as spectral inferences during the analysis. Acidic dilution with reagents containing nitric acid (HNO_3_) in different amounts together with other additives, such as ETDA or Triton X-100 and butanol, for the analysis of whole blood, serum, or urine is widely used [22, 42–45]. Few publications apply alkaline dilution using ammonia (NH_3_) mixtures with chelating additives or TritonX-100. This dilution is mostly exclusive for whole blood samples as it provides certain advantages for this matrix [22, 46–48]. Precipitation of proteins is minimized under alkaline conditions, and thereby, a clogging of the sample introduction parts of the instrument and memory effects is better controlled and minimized [46, 49]. Sample preparation procedures not only differ slightly between the specific matrices but also depend on the elements to be analyzed. Correct pH is essential for controlling the elements' solubility and avoiding memory effects during the instrumental analysis.

This work aimed to develop rapid and reliable methods for analyzing a broad range of toxic and essential elements in liquid human matrices, such as whole blood, serum, and urine. The setup aims to be straightforward, with the same or similar chemical and reagent composition used for all three matrices. The methods should allow an analysis of different matrices on the same day with various combinations of elements if needed and required for clinical diagnostic as well as biomonitoring.

## 2. Materials and Methods

Analytes of interest are toxic and potential toxic elements, such as silver (Ag), aluminum (Al), arsenic (As), barium (Ba), beryllium (Be), bismuth (Bi), cadmium (Cd), cerium (Ce), mercury (Hg), lead (Pb), palladium (Pd), platinum (Pt), antimony (Sb), strontium (Sr), tellurium (Te), thorium (Th), thallium (Tl), uranium (U), tungsten (W), and zirconium (Zr). In addition, essential and potential essential trace elements, such as boron (B), cobalt (Co), chromium (Cr), copper (Cu), iodine (I), lithium (Li), manganese (Mn), molybdenum (Mo), nickel (Ni), selenium (Se), tin (Sn), vanadium (V), and zinc (Zn) [3, 4].

### 2.1. Chemicals and Standards

2-propanol and nitric acid ≥65% of trace select quality were purchased from Fluka or Honeywell/Riedel de Haen (Bucharest, Romania), ammonia 25% of suprapure quality was purchased from Merck (KGaA, Darmstadt, Germany), and hydrogen peroxide 30% for ultratrace analysis, Triton™ X-100 for molecular biology, and Tergitol 15-S-9 were purchased from Sigma-Aldrich (Steinheim, Germany). Water was obtained from a Milli-Q-Advantage A10 ultrapure water purification system (Merck KGaA, Darmstadt, Germany). Gases, such as argon and helium, both of 5.0 quality were purchased from Linde (Linde Gas AS, Oslo, Norway). Single standards from Inorganic Ventures (Christiansburg, VA, USA) were used as internal standards (rhenium, Re, and rhodium, Rh), as a stabilizer (gold, Au), or for preparation of the spike mixtures (Al, B, Be, Bi, Ce, Pb, Sb, Te, Th, U, V, W, and Zr; Supplementary Material Tables S1 and S2). Standard spike solutions were prepared and stored at room temperature in 50 mL polypropylene (PP) tubes (Greiner bio-one GmbH, Kremsmünster, Austria). Weight control was performed and logged before and after usage to control for weight loss due to evaporation. Metrological traceability of standard solutions is given through certificates provided by the supplier, and documentation of storage conditions and further use by our laboratory.

### 2.2. Human Sample Material

ClinCal® whole blood, serum, and urine calibrators, which are lyophilized sample materials from Recipe (Recipe Chemicals and Instruments GmbH, Germany), were applied for matrix-matched calibration (Supplementary Material Table S3). Lyophilised ClinChek® (Recipe Chemicals and Instruments GmbH, Germany) and Seronorm (Sero AS, Billingstad, Norway) control samples of different concentration levels were used for method validation and later for internal quality control of performance with whole blood (levels I, II, and III), serum (levels I and II), and urine (levels I and II) samples together with human real samples from the Quebec Multielement External Quality Assessment Scheme (QMEQAS, Laboratoire de Toxicologie, Institut National de Sante Publique du Quebec, Canada). Lyophilised sample material was reconstituted in Milli-Q-water according to the given procedure. Aliquots were transferred to 1.5 mL PP-vials (Eppendorf, Hamburg, Germany) and stored at < −20°C until analysis. Metrological traceability of calibration and control material is documented through certificates provided by the supplier and documentation of preparation, storage conditions, and further use by our laboratory.

Real samples were provided from two different Norwegian studies. Whole blood and serum samples from the multicenter donor study comprised female and male blood donors from Tromsø (*N* = 17 + 14) collected in 2016 (REK 2015/541). Details on the sampling procedure were published previously [14]. Sample material was frozen in PP-vials at < −30°C until analysis [50]. Urine samples were from the MISA study covering pregnant and nonpregnant women from Northern Norway (*N* = 15 + 15) with a sampling period from 2017 to 2021 (REK 2017/816). MISA samples were stored frozen in PP-vials at −35°C in the biobank of UiT The Arctic University of Norway [50].

### 2.3. Sample Preparation

The samples were thawed overnight in the fridge at 3–8°C. Then, whole blood and serum samples were placed on an Intelli-mixer RM-2 (ELMI, Newbury Park, California) for mixing and tempering for at least 30 min, while urine samples were brought to room temperature for 30 min and vortexed well prior to sample preparation. An automated liquid handler Tecan Freedom Evo 200 (Männedorf, Switzerland) equipped with an eight-channel liquid handler arm (LiHa) for conductive disposable tips, a robotic manipulator arm (RoMa) for transport of microtiter plates, and a microplate shaker (BioShake, Quantifoil Instruments GmbH, Jena, Germany) was used for sample dilution. Disposable tips (50 *µ*L, 200 *µ*L, and 1000 *µ*L) used for the dilution process were of Tecan pure quality. Forty-eight samples per batch (including four calibration samples, four Milli-Q-water blanks, two control samples of each level and from both distributors, and 28 or 32 real samples depending on the matrix type) together with a 7.5 mL 48-well plate (Perkin Elmer, Waltham, MA, USA) were placed on the carriers of the liquid handler (Supplementary Material Figure S1).

**Figure 1:**
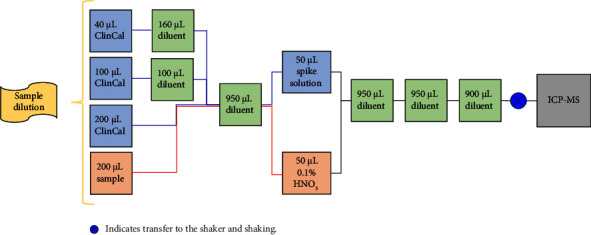
Workflow of the dilution procedure. Indicates transfer to the shaker and shaking.

All reagents (e.g., diluent and spike solutions) were placed onto the liquid handler right before their use, and the lids of the vials, which were used for a second or third round of sample preparation on the same day, were closed in between the preparation of the different batches. Sample dilution was performed as follows and displayed in Figure 1: 40 *µ*L (calibration sample 1), 100 *µ*l (calibration sample 2), and 200 *µ*L (calibration sample 3) of the ClinCal® calibrator sample, and 200 *µ*L of the regular samples were transferred to a 7.5 mL 48-well plate (Perkin Elmer, Waltham, MA, USA). 160 *µ*L and 100 *µ*L diluents consisting of Milli-Q water, 10% v/v ammonia and 2-propanol, 0.08% v/v Triton X-100 , and 0.25 *µ*g/L Au standard solution, were added to calibration samples 1 and 2, followed by addition of 950 *µ*L diluent to all wells. 50 *µ*L of three different spike mixtures were added to calibration sample 1 to 3 (Table S1), and 50 *µ*L 0.1% HNO_3_ was added to the regular samples to compensate for the minimal amount of HNO_3_ in the spike solutions added to the calibration samples. Furthermore, 2 × 950 *µ*L and 1 × 900 *µ*L diluent were added consecutively, and the samples were mixed by shaking after the final diluent addition. Sample preparation of 48 samples was achieved within less than 40 min.

### 2.4. Instrumental Analysis

Analysis of the selected elements was performed on a Perkin Elmer NexION 300D ICP-MS instrument (Perkin Elmer, Waltham, MA, USA) equipped with an ESI-FAST SC2DX autosampler (Elemental Scientific Inc., Omaha, Nebraska, USA). For sample introduction, a 2.5 mL sample loop was installed at FAST DXi Integrated valve (Elemental Scientific Inc., Omaha, Nebraska, USA). An aqueous carrier solution consisting of 0.1% v/v Triton X-100 pushed the sample through the sample loop and towards the nebulizer by the microperistaltic pump. After the sample loop and prior introduction to the PTFE-micro nebulizer (Perkin Elmer, Waltham, MA, USA) which was mounted to a quartz nebulizer chamber, the internal standard solution, consisting of 20 *µ*g/L ReRo (, 10% v/v ammonia , and 10% v/v 2-propanol, was introduced via a T-piece. Black/black and green/orange peristaltic pump tubes were applied for carrier and internal standard solution, respectively, with a pump speed of 3 rpm. Argon nebulizer gas was introduced at a flow rate of 0.95 to 1.1 L/min. Standard quartz injector and torch were used for sample transfer to the plasma with a plasma gas flow of 18 L/min. A nickel sample and a skimmer cone, together with an alumina hyper-skimmer cone, were installed at the interface. The MS was run in kinetic energy discrimination (KED) mode with low (4.8 mL) and high (5.7 mL) helium flow (Supplementary Material Table S4) and 1600 W RF generator power. Based on removing ClO interference by helium KED modes, <2% interference is still present with low helium flow, and respectively <0.5% interference is present with high helium KED flow. Measurements of the different elements were conducted in triplicates with 20 sweeps per reading, and individual adopted dwell and integration times as displayed in Supplementary Material Table S4.

### 2.5. Quantification

For quantification, the software generated a four-point calibration curve (NexION vs 1.5). Details on calibration concentrations can be found in the ESI Table S3. After performing the internal standard correction, linear regression through zero and a blank subtraction were applied.

### 2.6. Method Validation

The method was validated using internal quality assurance ClinCheck and Seronorm control samples as well as samples from the external QMEQAS round-robin test with different concentration levels in order to evaluate linearity, precision, repeatability, accuracy, intermediate precision, selectivity, robustness over time, high-throughput, and flexibility of the method. Procedure blank samples were included to control the background and calculate the method detection limits (MDLs) and method quantification limits (MQLs). Applicability for real samples was investigated using samples from different population studies from Northern Norway.

## 3. Results and Discussion

In the present work, a dilute-and-shoot method for 33 elements (i.e., Ag, Al, As, B, Ba, Be, Bi, Cd, Ce, Co, Cr, Cu, Hg, I, Li, Mn, Mo, Ni, Pb, Pd, Pt, Sb, Se, Sn, Sr, Te, Th, Tl, U, V, W, Zn, and Zr) in human whole blood, serum, and urine was developed, validated, and tested for application within clinical diagnostic and biomonitoring.

### 3.1. Sample Preparation and Instrumental Analysis Method

Prior to sample preparation, a mixing on a blender was tested to be sufficient for whole blood and serum samples. At the same time, vortexing was necessary for urine samples to dissolve elemental adsorption from the container wall and elemental precipitation. Underestimation of Ag, Cd, Hg, Pb, Se, and Sr was mainly observed when urine samples were not mixed properly (data not shown). Similar observations were described for urine samples previously [51].

For sample preparation, an alkaline dilution was chosen as the initial point to keep the reagent composition as similar as possible for all three matrices. Especially for whole blood and serum samples to a lesser extent, an alkaline media is preferred to promote the lysis of red blood cells and to avoid clogging of the sample introduction system by protein precipitation, where especially the injection valve (ESI) and the micronebulizer can be affected strongly. On the other side, also memory effects may be reduced by application of an alkaline solution. In addition, I requires alkaline conditions for reliable determination, since acidic conditions foster volatilization and memory effects of I during sample preparation and instrumental analysis [49, 52]. A previously published work compared acid digestion with an alkaline dilution for a broad group of elements and successfully demonstrated several advantages of the latter applied method [46]. Simple sample dilution is a fast and easy procedure and minimizes the contribution of background contamination from vessels used for microwave or other digestion processes. The need for lower sample volumes by alkaline dilution compared to acid digestion is attractive for saving the highly valuable sample material often stored at biobanks in very limited available amounts. Using less reagent solutions makes the sample preparation more cost-effective and attractive for high-throughput analysis in extensive biomonitoring studies and clinical diagnostics. However, a compromise between dilution of the sample matrix and sensitivity during instrumental analysis must be considered. As a starting point for method establishment and implementation, the methods developed by Michel [53–55] were used and further modified and adopted for a broader range of elements. As a first step, the sample preparation was adapted to an automated liquid handler, and the samples were prepared in 48-well plates as described previously in the Methods section. The composition of the diluent was slightly changed with the addition of small amounts of Au [56] and Triton X-100. By amalgamation, Au stabilizes elements, such as Hg, to prevent memory effects since Hg easily adheres to surfaces, such as autosampler tubes. The Au acts as a strong potent oxidizing agent that converts or maintains Hg as Hg-ion and keeps it in solution. Triton X-100 lyses blood cells and prevents adsorption of proteins on the sample introduction system [31–33]. Hence, Triton X-100 was added to the sample introduction rinse solution in higher concentration to prevent build-up of proteins between the sample injections and over time. To optimize for high-throughput analysis, save valuable sample material, and benefit from the 48-well format and its fully automated sample preparation, the NexION 300D instrument was modified with an integrated ESI microperistaltic pump and FAST DXi Integrated valve. This setup allowed a low sample introduction flow through a sample loop and an extra dilution by introduction of an internal standard solution via a T-piece mounted between the FAST DXi Integrated valve and the micronebulizer. Optimized higher dwell and integration times for the individual elements on the MS compensated for this additional online dilution during the sample introduction of the internal standard addition, keeping the sample volumes as low as possible.

Method development, validation, and implementation were performed over some years, starting with whole blood, serum, and urine, respectively. This allowed us to test and investigate different batches of solvents, reagents, disposable material, and other equipment. However, there arose a challenge with the 48-well plates during establishing the method for urine in late 2021. After a change to a new production batch of the 48-well plates occurred high background contamination of several elements, and especially for Cu. The high Cu contribution from the 48-well plates did not allow a correct quantification of Cu in urine samples due to high plate-to-plate and well-to-well variations of the contamination. This was not observed for other former production batches. A wash-step of the 48-well plates was introduced before sample preparation, where the wells of the plates were rinsed with 4 mL Milli-Q water containing 10% v/v of both ammonia and 2-propanol followed by 4 mL pure Milli-Q water. The plates were dried upside down overnight and used on the following day or when needed.

### 3.2. Blank Samples, Background Contribution, and Method Detection Limits

At the beginning of each sample run of the instrumental analysis, two Milli-Q water and diluent samples were analyzed for background contribution related to chemicals and equipment used for diluent preparation. It was evident that the multistream Xplorer pipette with its disposable tips (Eppendorf AG, Hamburg, Germany) used for the addition of ammonia and 2-propanol contributed to a minor background level of Cr in the diluent and the internal standard solution. In addition, diluent blanks (reagent blank samples) for monitoring the instrument baseline and instrumental carryover between the samples were run initially, after the calibration samples, after each 10th sample, or after an internal QA/QC sample set. Procedure blank samples were prepared with the other samples to control the overall background contribution. A contribution of Cu caused by the 8-channel liquid handler arm was observed. However, these contaminations affected the quality of the analysis only in a minor amount since Cu background contamination was stable over time, and Cu concentrations present in real samples are commonly much higher.

The procedure blank samples were also used to calculate the method detection limits (MDLs) and method quantification limits (MQLs). MDLs were set as three times the standard deviation of the concentrations of the procedure blank samples analyzed during the validation period of the individual sample matrix, while MQLs were set as ten times the standard deviation (Supplementary Material Table S5). For whole blood and serum, the MDLs covered a range from 0.003 *µ*g/L (Pt) to 7.08 *µ*g/L (Al) and 0.001 *µ*g/L (U) to 8.37 *µ*g/L (Al), respectively, with most of the elements showing sub-*µ*g/L MDLs (Tables 1, 2, 3, and 4 and Supplementary Material Table S5). Background contribution of Al reflected in higher MDLs is a common problem since Al is present in many disposal laboratory equipment and reagents [57]. Cu and Zn showed sub-mg/L MDLs. MDLs for urine were ranging from 0.004 *µ*g/L (W, Pt, Te, and Th) to 3.73 *µ*g/L (Cu) (Table 5 and 6 and Supplementary Material Table S5). With references to the already discussed Cu-MDL, its higher MDL is mainly related to newly introduced variability during the production of the newer batches of the 48-well plates. Previously published literature studies reported comparable [42, 45, 58–60] or slightly higher MDLs [21, 57, 59] by using similar ICP-MS instrumentation. Applications on expensive sector field ICP-MSs or newer generation instruments with novel MS/MS technology achieve lower MDLs [22, 61], which is not surprising. Other options, which may push down the MDLs but are not feasible at this time, are using clean room facilities for controlling ambient air contamination through volatile and particular bound elements, together with exclusive use of the instrument for background screening of the general population only [62].

### 3.3. Linearity

**Figure 2:**
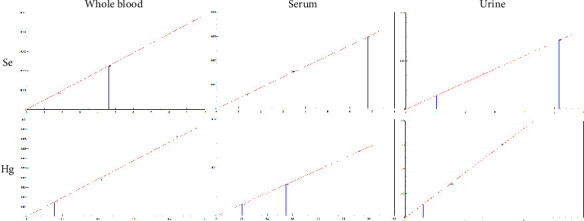
Examples of matrix-matched calibration curves prepared with ClinCal® calibrator samples for Se and Hg in whole blood, serum, and urine. Fluctuations of the triplicate measurements (relative standard deviation) were between 0.2% and 10%.

The linearity for all elements in the matrix-matched calibration curves and all three matrices was within the accepted specification with a regression coefficient (*R*^2^) of ≥0.999. Examples of matrix-matched calibration curves for selected elements, such as Se and Hg, are shown in Figure 2. By using the ClinCal® materials for the preparation and measurement of calibration curves, the highest calibration point is for several elements and matrices below the detected concentrations in the samples. The calibration curve is extrapolated further by the NexION software. An extrapolation works well for sample concentrations above the highest calibration point, as demonstrated by the measurements of the internal and external control samples, as the linear range of the detector on an ICP-MS instrument is over eight to more than ten orders of magnitude.

### 3.4. Whole Blood

#### 3.4.1. Accuracy

The accuracy of the established method was investigated over 14 months by preparing and analyzing sample sets of ClinChek and Seronorm controls on four different days (for Seronorm L-3 on three different days) as follows: ClinChek *N* = 6 + 6 + 6 + 11, Seronorm L-1 and L-2 *N* = 6 + 5 + 6 + 7 and Seronorm L-3 *N* = 6 + 6 + 7, and by different operators. Measured concentrations (mean concentration of all measurements) were compared with the assigned concentrations of these samples (Tables 1 and 2). General achievements for most elements and different sample materials show an acceptable deviation of ≤ 14%. All elements were within the assigned reference concentration ranges of the ClinChek materials, except for Mn and Hg in L-1 and Sb in all three levels. These were above the maximum reference concentrations (Table 1). Isobaric molecular interferences probably caused the high deviations for Sb in these ClinChek samples but were not observed in other later-used ClinChek batches (data not shown). However, much better performance was achieved for Sb in the Seronorm L-1 sample with a similar target concentration as in ClinChek L-1. Most elements were within the assigned reference ranges for Seronorm samples (Table 2). Exceptions were I and Cr (L-3 and L-1, respectively) with an observed overestimation, and Bi, Hg, Mo, and Tl (L-3) with concentrations slightly above the reference concentration ranges (Table 2). Concentrations in Seronorm samples were generally lower compared to the ClinChek materials, and reference concentrations were given as approximated reference concentrations for several targeted elements. The overestimations in the Seronorm material could be partly related to the inaccuracy of approximate reported target reference concentrations (i.e., Ag, Ce, and W), whereas the overestimation of I in the higher concentration range may be affected by some interferences, which are not removed using KED-mode with helium.

In addition to the ClinChek and Seronorm control samples, four fresh human samples from the Quebec Multielement External Quality Assessment Scheme were prepared and analyzed in triplicates. Results with deviation from the targeted concentration and coefficients of variation (CVs) for the triplicates are shown in Tables 7 and S6. Most elements show satisfactory performance with measured concentrations within the assigned concentration ranges. For Mn, an overestimation is observed for QM-B-Q1911 and QM-B-Q2001, which may be related to matrix interferences caused by large amounts of Fe present in the whole blood samples, where tailing from Fe-isotopes at m/z 54 and m/z 56 are affecting Mn measured at m/z 55 [32], which cannot be solved with KED-mode.

#### 3.4.2. Precision and Repeatability

Precision and repeatability of the established method were investigated by preparing and analyzing six replicates of ClinChek and Seronorm samples of different concentration levels (i.e., levels 1, 2, and 3) on the same day and by the same operator. The results are expressed as CVs and are given in Tables 1 and 2 for elements with assigned target concentrations and in Table S7 in the Supplementary Material for elements without given target concentrations. The intraday variation was far below 8% for most of the elements. Pt was an exception in the Seronorm samples, showing higher CVs due to targeted and measured concentrations very close to the MDL. Therefore, this element will not be discussed further for Seronorm samples.

The interday precision was investigated over a period of 14 months by preparing and analyzing sample sets of ClinChek and Seronorm controls on four different days (for Seronorm L-3 on three different days) as follows: ClinChek *N* = 6 + 6 + 6 + 11, Seronorm L-1 and L-2 *N* = 6 + 5 + 6 + 7, and Seronorm L3 *N* = 6 + 6 + 7, and by different operators. Results for the intermediate precision are expressed as interday CVs and are listed in Tables 1, 2, and S7. Most elements showed an interday variation below or far below 15% (Tables 1, 2, and S7). High and slightly higher observed CVs are mostly related to target concentrations close to the MDLs (i.e., Ag, Cd, and Pt; Table 2).

### 3.5. Serum

#### 3.5.1. Accuracy

The accuracy was investigated over a period of three to four months by preparing and analyzing sample sets of ClinChek and Seronorm controls on three different days (*N* = 8 + 8 + 8) and by other operators. Measured concentrations in ClinChek and Seronorm samples were within the assigned reference ranges (Tables 3 and 4). General achievements for most elements and different sample materials show deviations around and far below 10%. The target concentrations in Seronorm samples were generally lower compared to the ClinChek materials, and targeted concentrations were often given as approximated reference concentrations for several elements without given concentration ranges. Some over- and underestimations were observed for these elements, which could be partly related to the inaccuracy of approximate reported target reference concentrations (i.e., Ag, B, Cd, I, Th, V, and W).

Four fresh human serum samples from the Quebec Multielement External Quality Assessment Scheme were prepared and analyzed in triplicates. Results with deviation from the targeted concentration and CVs for the triplicates are listed in Table 8. CVs for elements without reference concentrations are given in Table S8. The measured concentrations were for all elements within the assigned concentration ranges, which is satisfactory.

#### 3.5.2. Precision and Repeatability

Precision and repeatability were investigated by preparing and analyzing eight replicates of ClinChek and Seronorm samples of concentration levels 1 and 2 on the same day and by the same operator. CVs are given in Tables 3 and 4 for elements with assigned reference concentrations and in Supplementary Material Table S9 for elements without assigned concentrations. Generally, the CVs for precision and repeatability were below and far below 8% for most of the elements (Tables 3, 4, and S9).

The interday precision was investigated over a period of three to four months by preparing and analyzing sample sets of ClinChek and Seronorm controls on three different days (*N* = 8 + 8 + 8) and by different operators. The results for the intermediate precision are listed in Tables 3, 4, and S9. Most elements generally showed an acceptable performance with CVs below and far below 10%. Exceptions were elements as B, As, Cd, Th, and W (Table 4), with higher CVs mostly related to lower sample concentrations.

### 3.6. Urine

#### 3.6.1. Accuracy

The accuracy was evaluated over a period of three months by preparing and analyzing sample sets of ClinChek and Seronorm controls on three different days with *N* = 8 + 8 + 8 (Seronorm L-2 *N* = 6 + 8 + 8) and by other operators. Measured concentrations were compared with the assigned concentrations of the internal control samples (Tables 5 and 6). Most of the measured concentrations in the ClinChek samples were within the assigned reference concentration range; exceptions were Al (in L-1 and L-2) and Hg (in L-2), with a slight underestimation (Table 5). Seronorm samples showed concentrations slightly above the upper reference concentration for Co, Hg, Ni, and Zn in L-1 material. For Mn, Pb, and Se, an under- or overestimation was observed at both levels (Table 6). However, urine performance within the ClinChek samples was much better than that of the Seronorm material.

Four fresh human urine samples from the Quebec Multielement External Quality Assessment Scheme were prepared and analyzed in triplicates. Results with deviation from the targeted concentration and CVs for the triplicates are listed in Table 9. Measured concentrations without given reference concentrations are shown in Supplementary Material Table S10. The measured concentrations for most elements were within the assigned concentration ranges, which is satisfactory. Exceptions were observed in sample QM-U-Q2106 for Zn and Bi with measurements below the lower reference concentration range.

#### 3.6.2. Precision and Repeatability

Precision and repeatability were investigated by preparing and analyzing eight replicates of ClinChek and Seronorm samples of concentration levels 1 and 2 on the same day and by the same operator. Tables 5 and 6 give an overview of CVs for intraday variability for elements with reference concentrations. Others are shown in the Supplementary Material Table S11. The CVs for precision and repeatability were generally below and far below 10% for most of the elements (Tables 5, 6, and S11), which is satisfactory.

The intermediate precision was investigated over a period of three months by preparing and analyzing sample sets of ClinChek and Seronorm controls on three different days with *N* = 8 + 8 + 8 (Seronorm L-2 *N* = 6 + 8 + 8) and of different operators. The results for the intermediate precision are listed in Tables 5, 6, and S11. Generally, many elements performed satisfactorily, with CVs around and below 10%. Exceptions were Ba, Be, Ce, Pt, and Zr in Seronorm samples with higher CVs, mostly related to target concentrations close to the MDLs (i.e., Al, Ce, Pt, and Zr; Table 6).

### 3.7. Robustness of the Method in All Three Matrixes

The robustness of the methods was investigated over the validation periods, during which different operators were involved in preparing the working solutions and samples, performing the instrumental analysis, and reprocessing the data. Containers for working solutions were filled up to an approximate final volume with Milli-Q water. This resulted in fluctuations in final total volumes for diluent, internal standard, carrier, and rinse solution from preparation to preparation. These variations in total volume and absolute concentrations of the reagents did not affect the results.

Currently, Triton X-100 is on the toxic compound list, and we wish to replace it with a suitable and less toxic chemical. Tergitol 15-S-9 was tested as a replacement in parallel runs on real samples, and the results were comparable to those of the analysis with Triton X-100.

A switch between different loop sizes with a 2.5 mL loop for analysis of the entire panel (33 elements) and a 1 mL loop for analysis of a minor number of elements, as for clinical analysis or particular specific needs within a research project, did not affect the results. This was systematically tested with our clinical analysis for Hg and Pb in 24 whole blood samples and Cu, I, Se, and Zn in 22 serum samples against the analysis of the entire panel. Regression analysis (Passing and Bablok and Deming) and its scatterplots did not show any introduced bias when switching between loop sizes and related autosampler methods (results not shown). Autosampler methods were customised for sampling time, loop filling time, delay time prior to MS analysis, and sample introduction time.

The overall results show the methods' robustness against minor changes while preparing working solutions and samples, as well as instrumental analysis and setup. The requirements for running and switching between different sample matrices, the number of elements and samples, and using the adopted instrumental setup to gain similar analysis quality are hereby shown.

### 3.8. Human Real Samples

#### 3.8.1. Whole Blood and Serum

Samples of female and male participants from the multicenter donor study were analyzed. Details on measured elements with average concentrations, concentration ranges, and detection frequencies are given in Table 10. Detection frequencies of the individual elements were similar for both genders. Elements such as Ag, B, Cd, Cr, Zr, and Pb had higher detection frequencies in whole blood since most of these elements have higher affinity to red blood cells, while Ba, Ce, Tl, and V had higher detection frequencies in serum. Similar patterns were reported by a previously published study investigating element distribution between plasma and cells [22]. Concentrations and concentration ranges differed slightly for B in whole blood, whereas males showed lower concentrations but higher concentrations for Ba and Pb than female participants. In serum, Ni and Ba were detected in higher concentrations in male participants. Multielement levels in whole blood or serum were published previously [21, 22, 42, 46–48, 63, 64]. One of the studies measured a broad range of elements in whole blood of healthy male blood donors and found similar concentrations for As, Cd, Co, Mn, Sn, and Zn. In contrast, concentrations were higher for Hg, Mo, Pd, Se, Sb, Sr, Mo, Pd, Pb, Tl, and Pb and lower for Ba compared to the present study [48]. Other work reported similar serum levels in healthy pregnant women for As and Sr, lower concentrations for Ba, Cd, Co, Ni, and Zn but higher for B, Cr, Cu, Li, Mn, Mo, Sb, and U [46]. A good agreement was observed for several and primarily essential elements, with one of the most comprehensive studies determining 73 elements in different human fluids from a German population [22]. Higher whole blood concentrations in the German population were observed for B, Bi, Cd, I, Mn, Pb, Sn, Sr, Tl, and U, while lower concentrations were found for As, B, Ce Cr, Hg, Pd, Sb, and V than that of the present study. In serum, higher concentrations were found in the German population for B, Cd, Sr, and Mo and lower for Ag, As, Ce, Co, Cr, Hg, Ni, Mn, Pd, Sb, and V than that of the Norwegian blood donors.

#### 3.8.2. Urine

Samples of nonpregnant and pregnant women from Northern Norway were analyzed. Details on measured elements with average concentrations, concentration ranges, and detection frequencies are given in Table 11. Detection frequencies were similar in nonpregnant and pregnant women for most of the analyzed elements, except for Cr, Hg, Sb, and Zr, which were detected more frequently in pregnant women. Cd and Pb showed higher detection frequency in nonpregnant women but lower median concentration and concentration ranges for Pb than that in pregnant women. Li and As concentrations were generally higher in nonpregnant women. Multielement levels in urine were reported in previously published studies [22, 40, 44, 45]. Exposure to alkali, alkali earth, and transition metals in urine samples from pregnant women from Western Australia was investigated. Similar urinary Sr concentrations as the pregnant women from Northern Norway were found, while other metals were reported in higher maximum concentrations in Australia [40]. Concentrations of toxic and essential elements in a population from central Ethiopia were generally higher than in the female population of the present study, except for the mean concentrations for Ag, As, Cu, Pb, and Sr [44]. Compared to the multielement study from Germany [22], maximum concentrations in the present study were lower for Ba, Cd, Cr, I, Li, Mo, Sn, Sr, Tl, and W, whereas mean concentrations were higher for most of these elements. Concentrations were not creatinine-adjusted in either of these studies. However, creatinine normalization must be performed for a reliable and correct interpretation of the results, especially for clinical diagnosis.

#### 3.8.3. Applicability for Clinical Diagnostic and Biomonitoring

Applicability of the methods for clinical diagnostic was investigated for selected toxic and (potential) essential elements in whole blood and serum. For urine, it is under progress, but since the present data are not creatinine adjusted, urine will not be discussed further here. The implemented method is suitable for clinical diagnostic as clinical reference concentrations are covered for the majority of the elements described in Table 12 and the National User Manual for Medical Biochemistry in Norway [65, 66]. Al and Cr showed higher MQLs related to significant background contribution during sample preparation, which makes it challenging to quantify these elements in the serum of nonexposed patients. For biomonitoring studies, it is a common approach to include concentrations between MQL and MDL in statistical modelling. In the case of Cr, as demonstrated for the real samples, the detection frequency was much higher in whole blood compared to serum due to the application of the MDLs. Other datasets generated in our laboratory achieved lower MDLs for Cr in serum (unpublished results), comparable to those of whole blood and urine presented here. For Al, background contribution is a known problem. Hence, a limitation of this method was discussed earlier in this publication. The choice of gloves might be a reason for this Al-background problem and must be investigated closely in the future. Background contribution, and especially batch-to-batch variability in the background contribution of the equipment and chemical reagents, clearly demonstrates the challenges for some specific elements in the present work. For clinical application, a limitation for Cr and Al is identified, as due to high MDLs, low concentrations cannot be reliably measured in serum (Table 9). Only moderate to high exposure can be diagnosed. Analysis of human real samples showed that the method allows measurements of 33 elements in at least one or all three matrices, making the method applicable for its purpose.

## 4. Conclusions

The method validation results showed good accuracy, precision, and reproducibility for most elements in all three matrices. The same reagent and instrumental setup were applicable for all three matrices. To our knowledge, this streamlined approach has not been demonstrated in previously published literature studies. It allows a rapid and easy whole blood, serum, and urine analysis within one day without changing the reagent composition or requiring long equilibration times. When switching between different matrices with different reagent setups, the risk of human error is minimized. Analyses of human real samples highlight that the presented methods allow accurate and reliable measurement of 33 elements in whole blood, serum, and urine at concentrations relevant to clinical diagnostics and biomonitoring. There is currently a limitation for Al and Cr when analyzing low concentration levels. This limitation needs follow-up and consideration for some changes in the setup during sample preparation and for the applied chemicals, reagents, and disposable equipment.

## Figures and Tables

**Table 1:** Accuracy, precision, and repeatability for analysis of ClinChek whole blood samples.

Element	MDLs	ClinChek L1 (lot 445)						ClinChek L2 (lot 455)						ClinChek L3 (lot 445)					
		Concentrations			Deviation	CVs (%)		Concentrations			Deviation	CVs (%)		Concentrations			Deviation	CVs (%)	
		Target	Range	Measured	(%)	Intraday	Interday	Target	Range	Measured	(%)	Intraday	Interday	Target	Range	Measured	(%)	Intraday	Interday
**Co**	0.001	1.53	1.14–1.91	1.49	−2.4	3.8	**4.1**	7.05	5.64–8.46	7.01	−0.6	3.7	**3.4**	13.1	10.5–15.7	13.1	0.3	1.2	**4.0**
**Ni**	0.057	1.93	1.54–2.31	1.93	−0.1	5.1	**18**	4.75	3.80–5.70	4.65	−2.1	3.5	**6.5**	12.9	10.4–15.5	12.5	−2.7	1.7	**3.7**
**Se**	0.503	76.7	61.4–92.1	78.1	1.8	2.7	**5.1**	126	101–151	126	0.0	2.9	**4.2**	169	135–203	171	1.3	2.9	**5.7**
**Sn**	0.112	2.04	1.63–2.45	1.8	−9.5	7.0	**6.7**	4.53	3.63–5.44	4.21	−7.0	1.4	**3.6**	9.22	7.38–11.1	8.20	−11	2.0	**4.9**
**Cr**	0.163	1.25	0.941–1.57	1.29	2.8	10	**14**	5.49	4.39–6.59	5.25	−4.3	2.0	**6.8**	10.9	8.74–13.1	9.87	−9.5	2.3	**4.3**
**Mn**	0.060	8.01	6.41–9.61	12.9	61	3.6	**4.9**	14.9	11.9–17.9	17.8	20	3.0	**4.8**	21.4	17.1–25.7	23.1	8.2	1.7	**5.0**
**Cu ** ^*∗*^	0.048	0.679	0.543–0.815	0.71	4.9	3.1	**4.4**	1.10	0.877–1.32	1.10	−0.1	3.1	**2.9**	1.67	1.34–2.00	1.68	0.3	1.6	**4.3**
**Zn ** ^*∗*^	0.001	4.68	3.75–5.62	4.69	0.3	2.5	**3.9**	6.42	5.14–7.71	6.46	0.7	2.4	**3.7**	8.21	6.57–9.85	8.33	1.5	1.8	**5.2**
**As**	0.047	5.25	4.20–6.30	5.30	0.9	4.3	**5.6**	10.1	8.11–12.2	9.87	−2.3	3.2	**5.8**	19.4	15.5–23.3	19.3	−0.6	2.9	**6.5**
**Mo**	0.030	2.17	1.74–2.61	2.52	16	7.6	**12**	4.66	3.73–5.59	5.25	13	5.4	**12**	8.79	7.03–10.5	10.1	15	0.8	**11**
**Ag**	0.009	1.89	1.52–2.27	1.76	−6.8	4.1	**4.8**	4.34	3.47–5.21	4.09	−5.7	4.3	**3.5**	8.91	7.12–10.7	8.07	−9.5	1.4	**4.1**
**Cd**	0.022	1.19	0.948–1.42	1.20	0.8	14	**14**	2.93	2.35–3.52	2.91	−0.6	8.6	**10**	6.40	5.12–7.68	6.42	0.4	6.0	**7.5**
**Sb**	0.035	1.00	0.803–1.20	4.03	303	2.6	**34**	3.74	2.99–4.49	6.03	61	4.7	**5.5**	9.10	7.28–10.9	11.8	30	4.0	**8.0**
**Pt**	0.003	1.67	1.33–2.00	1.61	−3.6	2.9	**2.9**	2.47	1.97–2.96	2.41	−2.4	2.1	**3.4**	4.96	3.97–5.95	4.83	−2.6	1.7	**4.5**
**Tl**	0.027	0.840	0.672–1.01	0.854	1.6	5.1	**4.2**	4.24	3.39–5.09	4.22	−0.6	2.4	**3.3**	8.55	6.84–10.3	8.49	−0.7	1.1	**3.7**
**Hg**	0.004	1.44	1.01–1.87	2.19	52	5.6	**14**	6.47	4.86–8.09	7.08	9.5	3.1	**12**	12.1	9.64–14.5	12.8	5.5	2.1	**12**
**Pb**	0.097	59.1	47.3–70.9	57.8	−2.3	2.5	**2.0**	228	182–274	223.0	−2.2	2.4	**2.4**	446	357–535	430	−3.5	1.1	**3.0**

Intraday variation coefficients (CVs) (*N* = 6) and interday CVs (*N* = 6 + 6 + 6 + 11). Method detection limits based on blank samples (*N* = 21). Concentrations in *µ*g/L; ^*∗*^concentrations in mg/L. With bold text for single elements and bold numbers for interday CVs.

**Table 2:** Accuracy, precision, and repeatability for analysis of Seronorm whole blood samples.

Element	MDLs	Sero L1 (lot 1406263)						Sero L2 (lot 1406264)						Sero L3 (lot 1509408)					
		Concentrations			Deviation	CVs (%)		Concentrations			Deviation	CVs (%)		Concentrations			Deviation	CVs (%)	
		Target	Range	Measured	(%)	Intraday	Interday	Target	Range	Measured	(%)	Intraday	Interday	Target	Range	Measured	(%)	Intraday	Interday
**Be**	0.141	<0.02		<0.141				5.17	4.13–6.22	4.87	−5.8	5.3	**5.3**	10.1	8.10–12.2	10.2	0.7	4.0	**5.4**
**V**	0.217	0.970	0.580–1.35	1.10	14	6.8	**15**	4.98	3.98–5.98	4.58	−8.0	1.9	**5.3**	4.40	3.50–5.30	4.52	2.8	5.1	**5.2**
**Co**	0.001	0.200	0.120–0.280	0.188	−6.2	5.3	**10**	5.18	4.13–6.22	5.42	4.6	1.8	**2.1**	10.3	8.30–12.4	10.9	5.8	0.9	**1.7**
**Ni**	0.057	1.38	1.10–1.66	1.59	25	6.4	**19**	15.9	12.7–19.1	15.2	−4.5	0.8	**2.0**	11.0	8.80–13.3	11.2	1.9	1.0	**2.6**
**Se**	0.503	60.0	48.0–72.0	60.1	0.1	1.8	**6.5**	161	128–193	164	1.8	1.5	**3.6**	198	158–238	225	13	1.8	**3.3**
**Sn**	0.112	0.180	0.150–0.220	0.164	−9.0	12	**19**	5.24	4.19–6.30	4.93	−5.9	1.8	**3.8**	9.90	7.90–11.9	10.8	8.8	1.7	**3.4**
**I**	0.193	28.6	22.8–34.3	30.0	4.7	2.7	**8.5**	107	86.0–129	126	18	2.3	**3.7**	165	132–199	253	53	0.9	**2.2**
**B**	3.91	180	appr	170	−5.4	4.1	**11**	158	appr	164	3.6	5.0	**20**	379	appr	470	24	4.2	**14**
**Al**	7.08	11.6	5.80–17.5	10.5	−9.2	21	**37**	68.9	55.0–82.7	60.3	−12	5.6	**19**	88.0	70.0–106	78.2	−11	2.9	**17**
**Cr**	0.163	0.450	0.270–0.630	0.675	50	6.0	**22**	10.7	8.50–12.8	9.73	−9.0	1.7	**2.3**	35.5	28.4–42.6	33.7	−5.2	1.1	**1.6**
**Mn**	0.060	18.4	14.7–22.1	20.2	10	2.4	**4.6**	31.4	25.1–37.7	30.6	−2.6	1.4	**2.6**	33.3	26.6–39.9	33.8	1.5	2.0	**2.4**
**Cu ** ^*∗*^	0.048	0.640	0.500–0.760	0.653	2.0	2.4	**5.6**	1.34	0.270–1.60	1.33	−0.5	1.1	**1.8**	2.08	1.66–2.50	2.31	11	0.5	**1.2**
**Zn ** ^*∗*^	0.001	4.30	3.40–5.20	4.59	6.8	0.9	**4.8**	7.10	5.70–8.50	8.06	14	0.4	**2.4**	8.06	6.44–9.68	9.15	13	0.5	**2.2**
**As**	0.047	2.40	1.90–2.90	2.02	−16	8.0	**9.6**	14.1	11.3–17.0	12.0	−15	2.9	**4.0**	27.3	21.8–32.7	25.0	−8.5	1.8	**3.9**
**Sr**	0.105	37.0	appr	39.0	5.4	0.9	**8.6**	36.0	appr	39.4	9.5	2.9	**7.5**	37.0	appr	46.0	24	2.2	**7.0**
**Zr**	0.027	0.320	appr	0.337	5.2	7.4	**11**	0.330	appr	0.364	10	5.8	**9.6**	0.280	appr	0.417	49	7.3	**7.8**
**Mo**	0.030	0.510	0.410–0.610	0.542	6.2	4.8	**15**	5.31	4.24–6.37	5.53	4.2	2.7	**11**	6.20	4.90–7.40	7.42	20	2.7	**11**
**Ag**	0.009	0.075	appr	0.123	64	32	**38**	0.073	appr	0.133	83	24	**28**	0.078	appr	0.187	140	16	**19**
**Cd**	0.022	0.280	0.170–0.400	0.286	2.3	24	**35**	5.01	4.00–6.02	5.42	8.1	4.1	**5.7**	9.90	7.90–11.9	11.1	12	5.3	**5.6**
**Sb**	0.035	1.33	1.06–1.60	1.23	−7.9	6.9	**15**	25.9	20.7–31.1	24.2	−6.6	1.9	**6.6**	21.9	17.5–26.3	21.5	−1.7	2.8	**5.5**
**Te**	0.337	<0.005		<0.337				<0.005		<0.337				<0.005		<0.337			
**Ba**	0.224	457	appr	368	−20	1.2	**20**	477	appr	372	−22	0.6	**19**	557	appr	559	0.3	0.5	**15**
**Ce**	0.010	0.088	appr	0.101	14	6.9	**19**	0.086	appr	0.108	25	7.3	**16**	0.093	appr	0.129	39	10	**9.6**
**W**	0.017	0.060	appr	0.065	8.3	13	**13**	0.047	appr	0.068	46	14	**15**	0.141	appr	0.246	75	3.9	**11**
**Pt**	0.003	0.005	appr	0.006	15	23	**58**	0.005	appr	0.007	26	59	**73**	0.006	appr	0.009	48	14	**52**
**Tl**	0.027	0.007	0.003–0.01	<0.027				10.2	8.10–12.2	12.1	19	1.1	**1.8**	25.2	20.1–30.2	33.1	31	0.6	**1.1**
**Hg**	0.004	1.48	1.18–1.77	1.59	7.15	1.6	**15**	17.0	13.6–20.4	19.1	12	0.4	**12**	22.4	17.9–26.9	28.3	26	0.8	**13**
**Pb**	0.097	9.90	7.90–11.9	11.6	16.8	0.4	**3.0**	337	269–405	354	5.0	0.3	**1.3**	362	289–434	418	15	0.5	**1.2**
**Bi**	0.029	<0.005		<0.029				5.00	3.99–6.00	5.81	16	1.3	**2.1**	39.6	31.6–47.6	52.5	33	0.9	**1.6**
**Th**	0.010	0.009	appr	<0.010				0.009	appr	<0.010				0.010	appr	<0.029			
**U**	0.006	0.180	appr	0.163	−10	3.9	**6.9**	0.180	appr	0.173	−3.7	2.0	**4.4**	0.120	appr	0.146	22	3.9	**5.5**

Intraday variation coefficients (CVs) (*N* = 6); interday CVs L-1 and L-2 (*N* = 6 + 5 + 6 + 7) and L3 (*N* = 6 + 6 + 7). Method detection limits based on blank samples (*N* = 21). Concentrations in *µ*g/L; ^*∗*^concentrations in mg/L; appr: approximately concentration. With bold text for single elements and bold numbers for interday CVs.

**Table 3:** Accuracy, precision, and repeatability for analysis of ClinChek serum samples.

Element	MDLs	ClinChek L1 (lot 544)						ClinChek L1 (lot 544)					
		Concentrations			Deviation	CVs (%)		Concentrations			Deviation	CVs (%)	
		Target	Range	Measured	(%)	Intraday	Interday	Target	Range	Measured	(%)	Intraday	Interday
**Li ** ^*∗*^	0.004	3.84	3.46–4.22	3.96	3.2	0.9	**2.2**	3.64	3.27–4.0	3.58	−1.7	0.4	**7.3**
**Be**	0.109	1.82	1.46–2.18	1.78	−2.2	6.8	**13**	2.01	1.61–2.42	1.76	−13	4.6	**14**
**V**	0.224	2.44	1.95–2.93	2.65	8.6	5.1	**6.3**	2.75	2.20–3.30	2.77	0.9	4.0	**7.5**
**Co**	0.005	1.74	1.39–2.09	1.62	−7.0	4.0	**4.8**	2.18	1.74–2.61	2.02	−7.4	1.6	**7.3**
**Ni**	0.176	4.00	3.20–4.80	4.06	1.6	3.4	**5.6**	2.20	1.54–2.86	1.82	−17	2.3	**8.1**
**Se**	1.16	66.1	52.9–79.3	70.9	7.3	4.8	**5.2**	123	98.1–147	124	1.1	3.1	**7.0**
**Sn**	0.035	1.19	0.952–1.43	1.24	4.1	10	**8.0**	2.47	1.97–2.96	2.00	−19	4.4	**7.2**
**I**	0.057	88.7	71.0–106	89.8	1.2	0.8	**7.5**	105	83.9–126	103	−1.9	0.6	**7.2**
**Al**	8.38	22.7	15.9–29.5	22.4	−1.4	22	**29**	16.5	11.0–21.4	15.4	−6.5	7.4	**23**
**Cr**	0.543	3.88	3.10–4.66	4.02	3.6	5.1	**6.7**	2.72	2.18–3.27	2.97	9.2	4.1	**14**
**Mn**	0.201							2.26	1.81–2.72	2.20	−2.7	5.8	**8.8**
**Cu ** ^*∗*^	0.015	0.801	0.681–0.921	0.811	1.2	2.5	**4.6**	1.06	0.902–1.22	1.07	0.9	1.9	**7.3**
**Zn ** ^*∗*^	0.011	1.32	1.12–1.52	1.32	0.3	0.9	**3.0**	0.737	0.626–0.848	0.747	1.4	0.8	**7.5**
**As**	0.052	9.87	7.90–11.8	9.66	−2.1	3.2	**4.1**	9.71	7.77–11.7	9.74	0.3	4.2	**7.5**
**Mo**	0.023	1.42	1.14–1.70	1.33	−6.3	3.1	**4.9**	2.16	1.51–2.81	1.92	−11	4.3	**16**
**Pd**	0.003	5.14	4.11–6.17	5.23	1.7	2.5	**2.5**	4.83	3.86–5.79	4.91	1.7	1.6	**7.3**
**Ag**	0.054	9.85	7.88–11.8	9.76	−0.9	2.1	**3.4**	4.97	3.98–5.97	4.95	−0.5	1.0	**7.0**
**Cd**	0.062	2.28	1.82–2.74	1.99	−13	6.5	**13**	1.87	1.50–2.25	1.96	5.0	9.5	**9.8**
**Sb**	0.079	7.06	5.65–8.47	7.19	1.9	2.8	**6.1**	8.59	6.87–10.3	7.51	−13	4.9	**8.7**
**Ba**	0.528	94.3	75.4–113	103	8.8	1.2	**17**	428	364–492	417	−2.6	0.8	**6.8**
**Pt**	0.018	8.89	7.11–10.7	8.66	−2.6	0.9	**4.1**	265	212–318	250	−5.6	0.7	**8.0**
**Tl**	0.004	2.47	1.98–2.96	1.84	−26	1.7	**2.7**	1.86	1.49–2.23	1.75	−5.7	1.8	**6.6**
**Hg**	0.020	1.54	1.23–1.85	1.41	−8.7	3.7	**5.3**	1.53	1.23–1.84	1.33	−13	2.1	**10**
**Bi**	0.019							0.999	0.799–1.20	0.894	−11	1.5	**7.9**

Measured concentration for control materials (*N* = 3 × 8). Intraday variation coefficients (CVs) (*N* = 8) and interday CVs (*N* = 8 + 8 + 8). Method detection limits based on blank samples (*N* = 33). Concentrations in *µ*g/L; ^*∗*^concentrations in mg/L. With bold text for single elements and bold numbers for interday CVs.

**Table 4:** Accuracy, precision, and repeatability for analysis of Seronorm serum samples.

Element	MDLs	Sero L1 (lot 1309438)						Sero L2 (lot 1304416)					
		Concentrations			Deviation	CVs (%)		Concentrations			Deviation	CVs (%)	
		Target	Range	Measured	(%)	Intraday	Interday	Target	Range	Measured	(%)	Intraday	Interday
**Li ** ^*∗*^	0.004	5.26	4.20–6.32	5.39	2.5	1.1	**2.6**	9.69	7.74–11.6	10.1	4.0	1.2	**2.7**
**Be**	0.109	0.010	appr	<0.109				0.010	appr	<0.109			
**V**	0.224	1.10	appr	1.61	46	5.3	**10**	1.10	appr	1.62	47	3.1	**7.6**
**Co**	0.005	1.12	0.670–1.57	1.06	−5.1	4.6	**4.6**	3.05	2.13–3.97	2.97	−2.7	4.5	**3.8**
**Ni**	0.176	5.64	3.38–7.90	5.23	−7.4	3.9	**3.6**	9.90	7.90–11.9	9.14	−7.7	2.7	**2.3**
**Se**	1.16	87.0	76.0–99.0	87.1	0.1	2.6	**3.4**	138	120–157	137	−0.4	3.9	**4.0**
**Sn**	0.035	0.250	appr	0.244	−2.3	18	**22**	0.250	appr	0.257	2.6	14	**17**
**I**	0.057	71.8	appr	54.6	−24	1.2	**4.1**	60.9	appr	47.4	−22	1.6	**4.9**
**B**	1.39	79.4	appr	60.7	−24	12	**37**	135	appr	30.8	−77	17	**45**
**Al**	8.38	46.1	36.9–55.4	47.7	3.4	7.1	**15**	117	94.0–141	120	2.4	9.4	**12**
**Cr**	0.543	2.18	1.30–3.05	1.67	−24	6.5	**14**	5.70	4.00–7.50	4.62	−19	1.9	**9.1**
**Mn**	0.201	9.90	7.90–11.9	9.62	−2.9	3.3	**5.4**	14.5	11.6–17.4	14.5	0.2	3.1	**4.7**
**Cu ** ^*∗*^	0.015	1.09	0.999–1.18	1.14	4.8	0.8	**2.2**	1.85	1.70–2.00	1.99	7.6	1.0	**2.3**
**Zn ** ^*∗*^	0.011	1.10	0.952–1.24	1.16	5.3	1.0	**2.4**	1.62	1.40–1.83	1.76	8.5	1.2	**2.7**
**As**	0.052	0.400	appr	0.33	−17	20	**31**	0.380	appr	0.371	−2.2	17	**27**
**Sr**	0.169	95.0	appr	97.0	2.1	2.3	**5.2**	110	appr	111	0.6	2.1	**6.0**
**Zr**	0.075	0.252	appr	0.223	−12	12	**13**	0.193	appr	0.181	−6.0	13	**17**
**Mo**	0.023	0.760	appr	0.774	1.9	6.9	**11**	1.21	appr	1.09	−10	5.3	**7.6**
**Pd**	0.003	0.005	<0.005	<0.003				0.005	<0.005	<0.003			
**Ag**	0.054	0.160	appr	0.236	47	7.6	**16**	0.220	appr	0.297	35	4.7	**16**
**Cd**	0.062	0.130	appr	0.195	50	13	**33**	0.140	appr	0.161	15	28	**45**
**Sb**	0.079	10.4	appr	10.6	2.4	2.9	**5.9**	15.0	appr	15.3	2.3	1.8	**5.5**
**Te**	0.365	0.010	<0.01					0.010	<0.01	<0.365			
**Ba**	0.528	190	appr	181	−4.7	1.5	**3.4**	139	appr	139	0.0	3.4	**7.9**
**Ce**	0.009	0.117	appr	0.105	−11	7.0	**11**	0.125	appr	0.128	2.1	4.2	**8.4**
**W**	0.005	0.058	appr	0.039	−33	20	**22**	0.065	appr	0.029	−55	12	**28**
**Pt**	0.018	0.009	appr	<0.018				0.016	appr	<0.018			
**Tl**	0.004	0.090	appr	0.103	15	7.1	**7.4**	0.108	appr	0.118	9.0	7.4	**9.7**
**Hg**	0.020	1.07	0.530–1.60	1.05	−2.2	3.3	**5.1**	2.05	1.44–2.67	1.81	−12	3.2	**5.4**
**Pb**	0.184	0.400	appr	0.444	11	4.9	**5.1**	0.660	appr	0.624	−5.5	3.6	**5.6**
**Bi**	0.019	0.025	appr	<0.019				0.005	appr	<0.184			
**Th**	0.010	0.016	appr	<0.010				0.021	appr	0.011	−47	13	**88**
**U**	0.001	0.302	appr	0.275	−8.9	2.7	**5.6**	0.359	appr	0.317	−12	3.3	**6.2**

Measured concentration for control materials (*N* = 3 × 8). Intraday variation coefficients (CVs) (*N* = 8) and interday CVs (*N* = 8 + 8 + 8). Method detection limits based on blank samples (*N* = 33). Concentrations in *µ*g/L; ^*∗*^concentrations in mg/L; appr: approximately concentration. With bold text for single elements and bold numbers for interday CVs.

**Table 5:** Accuracy, precision, and repeatability for analysis of ClinChek urine samples.

Element	MDLs	ClinChek L1 (lot 1227)						ClinChek L2 (lot 1227)					
		Concentrations			Deviation	CVs (%)		Concentrations			Deviation	CVs (%)	
		Target	Range	Measured	(%)	Intraday	Interday	Target	Range	Measured	(%)	Intraday	Interday
**Be**	0.512	0.057	0.04–0.073	<0.512				0.246	0.184–0.307	0.298	21	35	**33**
**V**	0.039	20.8	16.6–25	20.4	−2.1	1.9	**2.2**	50.9	40.7–61	49.9	−2.1	0.8	**1.4**
**Co**	0.044	2.03	1.63–2.44	1.98	−2.7	1.9	**2.8**	34.3	27.5–41.2	33.5	−2.4	1.1	**1.1**
**Ni**	0.178	3.24	2.59–3.88	3.11	−4.1	3.1	**3.8**	29.6	23.7–35.5	28.4	−4.0	0.8	**1.3**
**Se**	1.39	34.8	27.8–41.8	29.2	−16	3.4	**8.3**	89.3	71.4–107	73.6	−18	4.3	**7.0**
**Sn**	0.063	5.14	4.12–6.17	5.10	−0.8	3.0	**5.5**	10.0	8–12	10.3	2.9	1.1	**2.8**
**I**	0.175	120	96.1–144	123	2.4	1.4	**1.4**	496	397–595	520	4.9	1.0	**1.2**
**Al**	10.3	34.00	27.2–40.8	27.0	−20	12	**16**	87.1	69.7–105	66.4	−24	3.5	**8.7**
**Cr**	0.173	4.04	3.23–4.85	4.05	0.2	3.7	**3.7**	19.9	15.9–23.9	19.5	−1.9	3.2	**2.7**
**Mn**	0.088	8.32	6.65–9.98	7.84	−5.7	2.1	**3.9**	19.9	15.9–23.9	18.2	−8.6	2.5	**4.0**
**Cu**	3.73	56.6	45.2–67.9	54.7	−3.4	9.3	**11**	111	88.7–133	99.7	−10.2	4.4	**7.3**
**Zn**	3.26	206	164–247	201	−2.3	2.2	**2.5**	531	425–638	545	2.6	1.2	**1.6**
**As**	0.114	43.5	34.8–52.2	43.0	−1.2	2.0	**2.1**	82.3	65.9–98.8	74.8	−9.1	1.4	**3.5**
**Mo**	0.027	23.9	19.1–28.7	23.5	−1.6	1.3	**1.7**	99.3	79.5–119	101	1.5	0.7	**1.8**
**Pd**	0.020	1.64	1.15–2.14	1.58	−4.0	2.5	**3.8**	9.72	7.29–12.1	8.10	−17	1.2	**3.1**
**Cd**	0.153	2.47	1.98–2.96	2.63	6.4	6.2	**9.4**	14.3	11.4–17.2	14.2	−1.0	1.9	**4.1**
**Sb**	0.110	11.8	9.4–14.1	11.6	−1.3	2.2	**5.3**	47.5	38–57	46.6	−1.9	0.5	**2.1**
**Ba**	0.095	10.8	8.65–13	10.4	−3.3	1.5	**4.1**	51.3	41.1–61.6	48.5	−5.4	0.8	**3.0**
**Pt**	0.004	0.040	0.028–0.053	0.038	−4.9	7.9	**14**	0.113	0.085–0.142	0.106	−5.9	6.8	**10**
**Tl**	0.004	6.80	5.44–8.16	6.45	−5.1	0.9	**1.6**	17.7	14.2–21.2	14.8	−17	2.2	**3.1**
**Hg**	0.041	2.34	1.41–3.28	1.75	−25	2.7	**7.9**	19.1	12.4–25.7	11.1	−42	2.8	**8.6**
**Pb**	0.325	27.5	22–33	28.5	3.5	2.0	**3.3**	54.4	43.5–65.2	56.7	4.2	2.1	**3.7**

Measured concentration for control materials (*N* = 3 × 8). Intraday variation coefficients (CVs) (*N* = 8) and interday CVs (*N* = 8 + 8 + 8). Method detection limits based on blank samples (*N* = 49). Concentrations in *µ*g/L. With bold text for single elements and bold numbers for interday CVs.

**Table 6:** Accuracy, precision, and repeatability for analysis of Seronorm urine samples.

Element	MDLs	Sero L1 (1706877)						Sero L2 (1403081)					
		Concentrations			Deviation	CVs (%)		Concentrations			Deviation	CVs (%)	
		Target	Range	Measured	(%)	Intraday	Interday	Target	Range	Measured	(%)	Intraday	Interday
**Li**	0.071	5.20	appr	5.11	−1.8	5.1	**6.8**	100	appr	101	0.9	1.2	**2.7**
**Be**	0.512	<0.005		<0.512				5.20	4.10–6.20	4.18	−19	26	**27**
**V**	0.039	0.130	0.100–0.150	0.438	237	8.3	**13**	26.0	20.7–31.2	27.7	6.4	1.9	**3.5**
**Co**	0.044	0.190	0.150–0.230	0.236	24	3.6	**8.1**	10.1	8.10–12.2	10.6	4.7	1.0	**2.3**
**Ni**	0.178	1.00	0.790–1.20	1.22	22	5.0	**6.5**	40.7	32.5–48.8	39.8	−2.1	1.0	**1.6**
**Se**	1.39	10.5	8.40–12.6	14.1	34	19	**16**	71.7	57.3–86.1	96.8	35	5.3	**5.4**
**Sn**	0.063	2.40	1.90–2.80	2.75	15	6.3	**6.7**	48.3	38.6–58.1	44.8	−7.2	4.2	**8.6**
**I**	0.175	170	135–204	174	2.4	0.6	**2.1**	297	237–356	311	4.8	0.7	**4.1**
**Al**	10.3	7.50	6.00–9.00	<10.3				107	85.0–128	94.8	−11	5.7	**7.6**
**Cr**	0.173	7.70	6.20–9.30	8.64	12	2.3	**3.3**	30.1	24.0–36.1	27.0	−10	3.2	**4.7**
**Mn**	0.088	0.330	0.260–0.40	0.465	41	14	**19**	9.30	7.40–11.2	7.34	−21	14	**14**
**Cu**	3.73	26.0	24.0–28.0	26.1	0.2	2.9	**16**	56.3	44.9–67.6	60.8	7.9	1.5	**19**
**Zn**	3.26	171	136–205	215	26	1.3	**4.0**	1281	1023–1538	1071	−16	8.1	**10**
**As**	0.114	97.0	77.0–116	100	3.2	0.8	**2.1**	261	209–314	271	3.9	1.4	**3.2**
**Sr**	0.179	74.0	appr	76.3	3.2	2.2	**6.2**	120	appr	122	1.9	17	**3.8**
**Zr**	0.035							0.057	appr	0.067	17	26	**37**
**Mo**	0.027	17.0	13.6–20.4	18.6	9.5	2.2	**2.0**	48.0	appr	48.6	1.3	1.9	**2.1**
**Ag**	0.019	<0.002	appr	<0.019				<0.002	appr	<0.019			
**Cd**	0.153	0.062	0.049–0.074	<0.153				4.90	3.9–5.8	4.83	−1.5	9.0	**13**
**Sb**	0.110	2.30	1.90–2.80	2.38	3.3	6.6	**6.7**	103	72.0–133	106	3.3	1.7	**5.3**
**Te**	0.358	0.023	0.018–0.028	<0.358				25.6	20.4–30.8	22.5	−12	4.6	**6.5**
**Ba**	0.095	3.60	appr	4.24	18	9.2	**11**	50.0	appr	46.5	−8.0	24	**29**
**Ce**	0.009	0.013	appr	0.014	11	23	**34**	0.027	appr	<0.009			
**Pt**	0.004	0.007	appr	0.005	−23	25	**48**	0.006	appr	<0.004			
**Tl**	0.004	0.106	0.098–0.114	0.116	9.7	5.5	**6.2**	8.60	6.87–10.3	8.40	−2.4	3.2	**2.6**
**Hg**	0.041	1.11	0.88–1.33	1.58	43	7.2	**12**	44.0	35.2–52.9	50.1	14	2.1	**12**
**Pb**	0.325	1.51	1.20–1.81	2.25	49	3.9	**20**	80.1	64.0–96.2	61.9	−23	21	**19**
**Bi**	0.285	0.010	0.008–0.012	<0.285				21.7	17.3–26	16.4	−25	7.1	**6.0**
**Th**	0.004	<0.0005	appr	<0.004				0.002	appr	<0.004			
**U**	0.010	0.017	appr	0.018	3.6	15	**18**	0.029	appr	0.022	−24	10	**14**

Measured concentration for control materials (*N* = 3 × 8). Intraday variation coefficients (CVs) (*N* = 8) and interday CVs (*N* = 8 + 8 + 8). Method detection limits based on blank samples (*N* = 49). Concentrations in *µ*g/L; appr: approximately concentration. With bold text for single elements and bold numbers for interday CVs.

**Table 7:** Accuracy for external quality assurance whole blood samples with deviation from the targeted assigned concentration and variation coefficients of three prepared and measured aliquots.

Element	QM-B-Q1911					QM-B-1921					QM-B-Q2001					QM-B-Q2012				
	Assigned concentrations			Dev.	CV (%)	Assigned concentrations			Dev.	CV (%)	Assigned concentrations			Dev.	CV (%)	Assigned concentrations			Dev.	CV (%)
	Target	Range	Measured	(%)		Target	Range	Measured	(%)		Target	Range	Measured	(%)		Target	Range	Measured	(%)	
**Be**	3.87	2.75–4.99	3.75	−3.1	**7.2**	9.64	6.90–12.4	8.77	−9.0	**10**	7.34	5.45–9.23	7.50	2.2	**0.2**	6.20	4.55–7.85	5.78	−6.7	**0.7**
**V**	1.86	1.23–2.50	1.68	−9.7	**6.3**	2.31	1.55–3.06	2.21	−4.5	**5.3**	2.97	2.11–3.83	2.61	−12	**2.0**	2.24	1.58–2.90	2.13	−4.9	**1.3**
**Co**	5.80	4.68–6.93	5.70	−1.8	**4.1**	8.19	6.68–9.70	7.82	−4.5	**1.3**	2.85	2.27–3.43	3.01	5.5	**4.3**	3.68	2.98–4.38	3.58	−2.7	**0.9**
**Ni**	5.77	3.87–7.67	5.50	−4.6	**1.9**	10.3	7.64–13.0	9.91	−3.8	**1.0**	6.03	4.32–7.74	6.18	2.5	**7.4**	3.88	2.52–5.24	3.98	2.5	**0.8**
**Se**	797	661–934	726	−9.0	**3.9**	210	163–247	210	−0.2	**0.8**	196	151–241	194	−1.0	**0.7**	287	227–347	285	−0.5	**0.9**
**Sn**	9.34	6.88–11.8	8.39	−10	**2.9**	5.45	3.86–7.04	4.77	−13	**1.4**	9.12	6.91–11.3	7.79	−15	**2.4**	11.0	8.37–13.6	10.0	−8.7	**0.7**
**I**	84.6	65.4–104	78	−7	**5.7**	68.1	53.9–82.4	68.6	1	**2.5**	62.8	48.4–77.2	66.8	6	**0.2**	118	92.7–143	132	12	**0.2**
**Al**	230	178–281	202	−12	**5.1**	89.3	62.0–117	76.8	−14	**3.6**	64.4	41.0–87.7	62.7	−2.6	**6.1**	114	81.1–146	111	−3.0	**8.1**
**Cr**	0.868	0.000–1.86	0.672	−23	**9.0**	4.75	3.10–6.41	4.25	−11	**2.8**	4.89	3.40–6.38	4.51	−7.9	**6.8**	1.66	0.726–2.59	1.54	−7.2	**4.8**
**Mn**	10.9	7.92–13.9	14.9	37	**6.7**	29.4	22.7–36.0	29.0	−1.5	**2.9**	12.7	9.52–15.9	17.7	39	**3.4**	17.7	13.6–21.8	20.8	17	**0.5**
**Cu ** ^*∗*^	2.79	2.47–3.11	2.73	−2.1	**4.7**	1.39	1.21–1.57	1.36	−2.0	**0.7**	3.18	2.79–3.57	3.27	2.7	**5.6**	1.99	1.73–2.25	1.98	−0.3	**0.5**
**Zn ** ^*∗*^	5.34	4.35–6.33	5.33	−0.2	**4.2**	6.67	5.48–7.87	6.75	1.2	**0.5**	9.71	7.97–11.5	9.98	2.8	**1.6**	7.36	6.01–8.71	7.65	3.9	**1.2**
**As**	14.8	10.0–19.5	12.2	−17	**2.7**	24.2	18.1–30.3	22.7	−6.0	**2.3**	6.78	4.04–9.52	6.73	−0.8	**2.4**	9.62	6.33–12.9	8.84	−8.1	**1.6**
**Sr**	16.3	13.0–19.6	16.6	1.9	**6.8**	18.1	14.5–21.8	16.8	−7.0	**3.2**	18.3	15.3–21.3	17.2	−5.9	**4.9**	30.9	25.9–35.9	31.0	0.3	**3.7**
**Mo**	9.05	7.09–11.0	9.17	1.3	**4.3**	7.49	5.79–9.19	7.76	3.7	**1.7**	7.35	5.80–8.90	8.04	9.3	**0.7**	5.53	4.31–6.75	5.40	−2.4	**2.6**
**Ag**	0.389	0.000–0.819	0.403	3.5	**9.2**	0.530	0.077–0.982	0.556	4.9	**4.0**	4.65	3.29–60.1	4.42	−5.0	**1.4**	3.29	2.30–4.28	2.36	−28	**1.7**
**Cd**	13.3	11.1–15.4	13.0	−2.4	**1.0**	1.89	1.41–2.37	1.94	2.7	**15**	3.51	2.86–4.16	3.34	−5.0	**5.9**	4.69	3.88–5.50	4.69	0.0	**8.2**
**Sb**	6.54	5.23–7.85	5.35	−18	**3.8**	15.2	12.4–18.0	14.3	−6.1	**4.8**	12.3	10.0–14.6	10.0	−19	**1.0**	4.45	3.55–5.35	4.16	−6.6	**3.8**
**Te**	6.76	4.93–8.59	5.98	−12	**5.8**	8.15	6.20–10.1	8.01	−1.7	**4.4**	4.13	2.65–5.61	4.16	0.7	**5.4**	9.95	7.45–12.5	9.68	−2.7	**1.9**
**Ba**	1.07	0.42–1.71	0.803	−25	**5.6**	1.34	0.649–2.03	1.11	−18	**6.0**	2.37	1.70–30.4	2.63	11	**14**	6.88	5.31–8.45	5.72	−17	**5.5**
**Pt**	1.91	1.42–2.39	1.68	−12	**4.9**	4.02	3.08–4.96	3.85	−4.3	**0.8**	1.12	0.833–1.41	1.10	−1.8	**2.9**	2.66	2.05–3.27	2.60	−2.3	**1.3**
**Tl**	5.44	4.73–6.15	5.64	3.6	**4.6**	3.56	3.07–4.04	3.63	2.1	**1.6**	2.06	1.72–2.40	2.09	1.5	**1.1**	3.60	3.05–4.15	3.63	0.8	**2.0**
**Hg**	38.9	30.6–47.3	37.0	−4.8	**4.6**	3.43	2.46–4.40	3.46	1.0	**2.9**	33.1	26.1–40.1	38.0	15	**3.8**	20.5	16.1–24.9	24.5	20	**2.5**
**Pb**	452	394–509	468	3.6	**4.1**	663	580–746	705	6.3	**0.4**	349	3.10–397	360	3.1	**1.0**	66.4	56.4–76.4	68.5	3.2	**1.8**
**Bi**	9.9	7.84–11.9	9.27	−6.0	**3.5**	8.13	6.49–9.77	7.78	−4.3	**1.7**	7.56	6.12–9.00	7.14	−5.5	**0.4**	13.6	11.1–16.1	12.7	−6.8	**1.5**
**Th**	2.01	1.37–2.66	1.81	−10	**3.2**	2.25	1.54–2.96	2.18	−3.0	**0.9**	4.08	2.90–5.26	3.88	−5.0	**1.1**	3.27	2.30–4.24	2.87	−12	**1.7**
**U**	0.831	0.704–0.957	0.804	−3.3	**5.1**	0.102	0.065–0.139	0.099	−3.1	**3.4**	0.069	0.041–0.096	0.067	−2.1	**2.6**	0.169	0.125–0.213	0.158	−6.7	**5.2**

Concentrations in *µ*g/L; ^*∗*^concentrations in mg/L. With bold text for single elements and bold numbers for CVs.

**Table 8:** Accuracy for external quality assurance programme serum samples with deviation from the targeted assigned concentration and variation coefficients of three prepared and measured aliquots.

Element	QM-S-Q1925					QM-S-Q2025					QM-S-Q2116					QM-S-Q2117				
	Assigned concentrations			*Dev.*	CV (%)	Assigned concentrations			*Dev.*	CV (%)	Assigned concentrations			*Dev.*	CV (%)	Assigned concentrations			*Dev.*	CV (%)
	Target	Range	Measured	*(%)*		Target	Range	Measured	*(%)*		Target	Range	Measured	*(%)*		Target	Range	Measured	*(%)*	
**Be**	24.3	17.7–31	25.6	5.4	**2.9**	12.7	10.1–15.3	13.4	5.3	**5.0**	9.40	7.37–11.4	10.8	15	**4.7**	4.55	3.38–5.72	5.01	10	**2.2**
**V**	0.698	0.409–0.987	0.935	34	**6.2**	n.a.		0.404		**12**	0.442	0.187–0.697	0.670	52	**6.8**	1.33	0.812–1.85	1.55	17	**4.9**
**Co**	4.08	3.25–4.92	4.08	−0.1	**0.1**	1.42	1.12–1.72	1.42	0.0	**4.7**	3.26	2.74–3.78	3.20	−1.8	**1.4**	6.51	5.59–7.43	6.57	0.9	**2.0**
**Ni**	5.04	2.6–7.47	4.89	−3.0	**1.4**	6.20	4.26–8.14	6.8	9.3	**2.1**	7.00	4.93–9.07	7.42	6.0	**2.5**	15.5	11.9–19.1	16.0	3.4	**0.3**
**Se**	325	271–380	344	5.7	**0.2**	126	99.9–152	129	2.2	**3.5**	261	214–308	253	−2.9	**3.1**	182	147–217	177	−2.8	**1.6**
**Sn**	4.43	3.03–5.83	4.43	−0.1	**2.5**	5.72	4.36–7.08	6.00	5.0	**3.5**	3.58	2.66–4.5	3.46	−3.4	**6.9**	3.19	2.34–4.04	3.16	−1.1	**3.6**
**I**	78.7	62.8–94.6	76.4	−2.9	**1.5**	108	80.2–136	105	−2.6	**0.9**	127	95.7–158	126	−0.5	**0.3**	65.1	45.1–85.1	64.7	−0.7	**1.4**
**Al**	23.1	15.5–30.7	20.8	−10	**8**	144	111–177	137	−4.6	**4.2**	82.9	62.8–103	80.9	−2.4	**2.4**	35.9	26.0–45.8	36.4	1.3	**13**
**Cr**	1.83	0.655–2.99	2.35	28	**3.7**	1.52	0.946–2.09	1.49	−2.2	**6.7**	3.16	2.25–4.07	2.86	−9.3	**4.7**	0.948	0.488–1.41	0.932	−1.7	**5.2**
**Mn**	2.57	1.90–3.25	2.79	8.5	**8.6**	1.09	0.629–1.55	1.36	24	**12**	2.08	1.45–2.71	2.33	12	**7.5**	4.12	3.14–5.10	4.17	1.2	**0.9**
**Cu ** ^*∗*^	1.42	1.24–1.6	1.53	7.5	**0.8**	1.19	0.984–1.4	1.21	1.5	**0.2**	1.35	1.12–1.58	1.35	−0.2	**0.6**	2.37	2.02–2.72	2.40	1.2	**0.5**
**Zn ** ^*∗*^	1.76	1.41–2.11	1.92	9.3	**0.2**	1.95	1.61–2.29	2.03	4.2	**0.6**	1.46	1.19–1.73	1.53	5.1	**1.4**	1.22	0.99–1.45	1.29	6.0	**0.7**
**As**	22.3	17.2–27.5	25.3	14	**0.8**	25.3	19.4–31.2	24.5	−3.3	**0.6**	11.9	8.60–15.2	11.3	−4.7	**9.2**	5.88	3.76–8.00	5.77	−1.8	**2.6**
**Mo**	4.26	2.87–5.65	4.07	−4.5	**1.7**	6.66	5.28–8.04	6.69	0.5	**1.8**	3.00	2.31–3.69	3.10	3.2	**3.0**	1.47	1.07–1.87	1.52	3.7	**1.0**
**Ag**	3.02	1.87–4.17	3.56	18	**2.0**	3.92	2.75–5.09	4.14	5.6	**1.1**	3.05	2.09–4.01	3.25	6.6	**1.9**	0.861	0.527–1.19	0.844	−2.0	**3.1**
**Cd**	0.597	0.269–0.925	0.523	−12	**27**	0.497	0.325–0.669	0.388	−22	**7.8**	2.46	2.00–2.92	2.47	0.3	**12**	10.5	8.83–12.2	10.7	2.0	**4.5**
**Sb**	2.63	1.95–3.31	2.50	−4.9	**2.1**	1.70	1.30–2.10	1.53	−10	**18**	1.97	1.52–2.42	1.99	1.2	**4.2**	1.37	1.03–1.71	1.33	−3.3	**14**
**Te**	2.96	1.46–4.46	2.80	−5.4	**7.8**	5.22	3.69–6.75	4.67	−11	**12**	8.96	6.50–11.4	7.72	−14	**5.7**	3.76	2.61–4.91	3.85	2.3	**5.8**
**Ba**	6.7	5.16–8.24	7.15	6.7	**1.4**	12.1	9.77–14.4	11.7	−3.4	**3.2**	14.8	12.0–17.6	14.9	0.3	**0.1**	2.55	1.77–3.33	2.49	−2.4	**3.7**
**Pt**	0.207	0.094–0.32	0.194	−6.4	**13**	0.791	0.597–0.985	0.762	−3.7	**1.8**	0.239	0.135–0.343	0.223	−6.9	**10**	0.521	0.374–0.668	0.545	4.6	**3.5**
**Tl**	1.89	1.53–2.25	2.08	10	**2.0**	1.08	0.863–1.30	1.12	4.1	**1.5**	2.57	2.09–3.05	2.73	6.3	**1.2**	1.52	1.22–1.82	1.58	4.1	**3.2**
**Hg**	1.84	1.34–2.35	1.69	−8.2	**3.3**	1.38	0.783–1.98	1.36	−1.2	**4.2**	1.88	1.14–2.62	1.62	−14	**2.4**	3.62	2.41–4.83	3.41	−5.9	**3.5**
**Pb**	31.3	23.3–39.2	32.2	2.8	**1.6**	40.1	32.6–47.6	39.4	−1.8	**0.6**	29.2	23.3–35.1	31.5	8.0	**2.8**	121	100–142	133	9.6	**0.8**
**Bi**	16	12.6–19.3	16.7	4.3	**0.8**	2.09	1.58–2.60	2.10	0.6	**0.4**	16.8	13.4–20.2	18.52	10	**1.1**	4.51	3.52–5.50	4.89	8.4	**1.6**
**Th**	1.31	0.896–1.73	1.41	7.3	**2.0**	3.39	2.72–4.06	3.20	−5.6	**0.6**	0.758	0.578–0.938	0.765	1.0	**0.3**	1.75	1.37–2.13	1.75	0.3	**0.6**
**U**	0.293	0.193–0.392	0.295	0.8	**1.2**	0.748	0.593–0.903	0.707	−5.5	**0.9**	0.207	0.151–0.263	0.210	1.6	**2.5**	0.469	0.367–0.571	0.482	2.7	**1.4**

Concentrations in *µ*g/L; ^*∗*^concentrations in mg/L. With bold text for single elements and bold numbers for CVs.

**Table 9:** Accuracy for external quality assurance programme urine samples with deviation from the targeted assigned concentration and variation coefficients of three prepared and measured aliquots.

Element	QM-U-Q2106					QM-U-Q2114					QM-U-Q2123					QM-U-Q2124				
	Assigned concentrations			Dev.	CV (%)	Assigned concentrations			Dev.	CV (%)	Assigned concentrations			Dev.	CV (%)	Assigned concentrations			Dev.	CV (%)
	Target	Range	Measured	(%)		Target	Range	Measured	(%)		Target	Range	Measured	(%)		Target	Range	Measured	(%)	
**Li**	211	172–250	211	−0.1	**0.9**	37.2	29.4–45	37.0	−0.6	**2.1**	263	219–307	277	5.0	**1.4**	436	365–507	490	11	**0.6**
**Be**	4.05	3.17–4.93	4.36	7.7	**26**	2.86	2.18–3.54	2.59	−9.4	**29**	6.16	4.93–7.39	5.62	−8.8	**11**	13.6	11.1–16.1	14.3	5.0	**21**
**V**	5.95	4.00–7.90	6.63	10	**1.8**	0.638	0.000–1.35	1.14	44	**4.5**	19.2	14.2–24.2	21.0	8.5	**1.8**	12.5	9.06–15.9	14.6	15	**0.9**
**Co**	5.06	4.28–5.84	5.06	0.1	**0.7**	0.409	0.251–0.567	0.385	−6.2	**11**	2.72	2.26–3.18	2.75	1.1	**1.2**	4.40	3.71–5.09	4.77	7.8	**1.7**
**Ni**	5.16	3.37–6.95	4.91	−5.0	**2.3**	2.16	0.826–3.49	2.05	−5.3	**1.2**	29.1	23.7–34.5	30.0	3.0	**0.3**	23.0	18.5–27.5	24.9	7.7	**1.4**
**Se**	101	68.0–134	113	11	**1.4**	75.4	48.3–103	83.7	10	**1.0**	78.0	50.2–106	87.1	10.5	**1.6**	193	139–247	240	20	**0.5**
**Sn**	10.4	8.63–12.2	8.79	−18	**10**	0.559	0.297–0.821	0.421	−33	**16**	145	123–167	138	−4.8	**0.6**	87.3	73.8–101	84.2	−3.6	**2.8**
**I**	94.5	74.2–115	98.0	3.6	**1.0**	85.1	66.7–104	89.2	4.6	**1.0**	168	134–202	184	8.8	**1.3**	317	254–380	360	12	**0.4**
**Al**	90.6	68.3–113	78.0	−16	**9.6**	68.0	50.1–85.9	60.6	−12	**16**	78.9	59–98.8	74.5	−5.9	**4.8**	107	81.8–132	111	3.3	**6.6**
**Cr**	35.2	28.5–41.9	35.5	1.0	**1.3**	1.50	0.864–2.14	1.74	14	**3.0**	30.4	24.6–36.2	32.4	6.3	**1.4**	6.19	4.72–7.66	6.68	7.3	**2.3**
**Mn**	1.27	0.099–2.44	1.02	−25	**7.4**	1.17	0.011–2.33	1.17	−0.3	**9.2**	8.60	6.38–10.8	8.02	−7.2	**3.4**	3.73	2.21–5.25	3.42	−9.0	**7.2**
**Cu**	469	403–535	415	−13	**0.4**	360	309–411	336	−7.2	**0.8**	82.6	69.1–96.1	79.8	−3.5	**0.4**	284	244–324	289	1.8	**1.6**
**Zn**	520	428–612	419	−24	**4.2**	371	302–440	340	−9.2	**1.8**	835	699–971	804	−3.9	**4.0**	297	240–354	295	−0.8	**2.3**
**As**	39.8	30.9–48.7	40.4	1.4	**1.2**	25.8	19–32.6	26.9	4.0	**3.7**	34.2	26.1–42.3	35.2	2.8	**1.1**	518	434–602	577	10	**0.5**
**Sr**	150	89.9–210	136	−10	**6.8**	177	106–248	176	−0.6	**0.3**	165	98.7–231	185	11	**6.8**	159	95.1–223	182	13	**4.3**
**Mo**	46.9	39.4–54.4	48.5	3.2	**1.8**	37.3	31–43.6	38.8	3.8	**0.4**	41.2	34.4–48	44.5	7.5	**1.7**	94.5	80.8–108	107	12	**0.4**
**Ag**	2.67	1.11–4.23	2.48	−7.7	**5.6**	0.807	0.234–1.38	0.636	−27	**10**	2.99	1.31–4.67	3.89	23	**1.5**	1.77	0.716–2.82	2.01	12	**3.0**
**Cd**	9.32	7.96–10.7	8.40	−11	**4.0**	0.778	0.511–1.04	0.838	7.1	**21**	7.56	6.43–8.69	7.71	1.9	**3.1**	0.95	0.662–1.24	1.14	17	**14**
**Sb**	1.27	1.06–1.48	1.30	2.1	**16**	0.233	0.148–0.318	0.248	5.9	**7.4**	2.34	1.99–2.69	2.35	0.3	**5.0**	3.45	2.96–3.94	3.66	5.7	**1.5**
**Te**	12.0	8.83–15.2	10.4	−16	**5.1**	0.652	0.164–1.14	0.570	−14	**47**	34.7	26.1–43.3	34.7	0.1	**0.6**	17.6	13.0–22.2	17.1	−3.2	**2.0**
**Ba**	8.52	7.20–9.84	7.90	−7.3	**8.0**	6.70	5.07–7.07	6.34	−5	**2.0**	33.9	29–38.8	36.7	8.3	**11**	5.33	4.46–6.20	5.46	2.5	**9.4**
**Pt**	1.69	1.33–2.05	1.44	−17	**2.8**	0.631	0.454–0.808	0.574	−10	**2.4**	2.730	2.23–3.23	2.71	−0.7	**1.8**	2.17	1.76–2.58	2.15	−0.9	**0.6**
**Tl**	6.37	5.43–7.31	5.92	−7.7	**0.6**	0.413	0.307–0.519	0.363	−14	**0.3**	28.6	24.5–32.7	27.7	−3.1	**0.4**	61.3	52.6–70.0	61.3	0.0	**1.3**
**Hg**	65.3	40.7–89.9	59.8	−9.1	**2.5**	5.68	2.49–8.87	5.36	−6.0	**2.5**	83.3	52–115	106	21	**0.9**	145	91.0–199	182	20	**0.8**
**Pb**	168	144–192	147	−13	**6.8**	4.12	3.01–5.23	4.61	12	**5.8**	652.0	559–745	708	8.6	**9.0**	132	113–151	135	2.5	**5.7**
**Bi**	4.44	3.44–5.44	2.85	−56	**2.4**	0.593	0.354–0.832	0.492	−21	**8.5**	8.34	6.54–10.1	7.44	−12	**3.6**	37.9	30.2–45.6	32.7	−16	**2.9**
**Th**	0.148	0.078–0.218	0.101	−46	**5.9**	0.103	0.055–0.151	0.072	−42	**4.1**	0.17	0.096–0.25	0.139	−24	**17**	0.291	0.167–0.42	0.237	−23	**6.9**
**U**	0.777	0.621–0.933	0.648	−20	**3.1**	0.100	0.072–0.127	0.092	−8.7	**3.9**	1.32	1.07–1.57	1.32	−0.4	**0.6**	2.86	2.32–3.40	3.030	5.6	**2.1**

Concentrations in *µ*g/L. With bold text for single elements and bold numbers for CVs.

**Table 10:** Concentrations in selected whole blood and serum samples from blood donors from Tromsø (*N* = 17 + 14).

Element	Whole blood						Serum					
	Females			Males			Females			Males		
	Concentrations		Detected	Concentrations		Detected	Concentrations		Detected	Concentrations		Detected
	Mean	Range	(%)	Mean	Range	(%)	Mean	Range	(%)	Mean	Range	(%)
**Li ** ^*∗*^							0.005	<0.004–0.005	6	<0.004		0
**Be**	0.244	<0.141–0.259	12	<0.141		0	<0.109		0	<0.109		0
**V**	0.292	<0.217–0.306	12	0.228	<0.217–0.230	14	0.473	0.385–0.550	100	0.516	0.392–0.666	100
**Co**	0.302	0.124–0.744	100	0.262	0.134–0.670	100	0.370	0.116–1.06	100	0.285	0.106–0.780	100
**Ni**	0.484	0.252–0.710	100	0.493	0.391–0.600	100	0.549	0.346–1.20	100	1.12	0.397–9.38	100
**Se**	91.8	73.5–113	100	102	71.6–158	100	85.1	56.2–114	100	90.1	64.5–133	100
**Sn**	0.300	<0.112–1.22	82	0.200	<0.112–0.546	86	0.178	<0.035–1.08	94	0.170	<0.035–0.430	93
**I**	32.1	21.9–47.9	100	31.1	21.8–37.2	100	58.1	45.5–78.0	100	54.8	40.9–79.1	100
**B**	31.0	11.9–72.3	100	19.2	6.53–36.4	100	19.2	<1.39–43.6	53	23.3	<1.39–37.8	36
**Al**	<7.08		0	<7.08		0	271	<8.38–271	6	370	<8.38–370	7
**Cr**	0.417	<0.163–0.544	82	0.347	0.184–0.496	100	0.607	<0.543–0.662	12	0.592	<0.543–0.608	29
**Mn**	15.4	12.2–19.1	100	15.3	11.8–19.6	100	0.802	0.600–1.03	100	0.770	0.537–1.03	100
**Cu ** ^*∗*^	0.948	0.711–1.44	100	0.845	0.713–1.11	100	1.24	0.856–2.03	100	1.12	0.787–1.95	100
**Zn ** ^*∗*^	5.89	4.48–6.96	100	5.96	5.30–6.77	100	0.99	0.825–1.34	100	0.925	0.780–1.08	100
**As**	4.61	0.412–12.8	100	4.68	0.331–13.9	100	2.99	0.227–13.3	100	3.88	0.329–15.1	100
**Sr**	14.3	5.88–19.2	100	15.3	8.84–42.0	100	26.5	11.6–36.2	100	27.7	18.4–80.1	100
**Zr**	0.038	<0.028–0.044	18	0.045	<0.028–0.064	50	<0.075		0	<0.075		0
**Mo**	0.552	0.307–0.784	100	0.566	0.356–1.22	100	0.855	0.452–1.85	100	0.782	0.458–1.28	100
**Pd**	0.032	<0.030–0.034	24	0.033	<0.030–0.033	14	0.008	<0.003–0.016	24	0.009	<0.003–0.011	21
**Ag**	0.089	0.018–0.246	100	0.100	<0.009–0.203	93	0.166	<0.054–0.349	71	0.132	<0.054–0.254	71
**Cd**	0.303	0.028–0.999	100	0.275	0.051–0.809	100	0.086	<0.062–0.115	18	0.078	<0.062–0.081	14
**Sb**	3.10	2.86–3.46	100	3.14	2.79–3.37	100	1.44	1.10–2.07	100	1.27	1.06–1.55	100
**Te**	0.347	<0.337–0.347	6	<0.337		0	<0.365		0	0.402	<0.365–0.402	7
**Ba**	0.645	<0.224–2.10	88	1.76	<0.224–14.7	86	1.61	1.06–3.62	100	4.31	1.05–42.4	100
**Ce**	0.024	<0.006–0.069	76	0.032	<0.006–0.122	86	0.037	0.019–0.110	100	0.030	0.023–0.042	100
**W**	<0.017		0	0.106	<0.017–0.106	7	0.009	<0.005–0.010	12	0.007	<0.005–0.008	21
**Pt**	0.006	<0.003–0.007	12	<0.003		0	0.041	<0.018–0.041	6	<0.018		0
**Tl**	0.036	<0.027–0.047	47	0.031	<0.027–0.036	50	0.020	0.005–0.028	100	0.018	0.008–0.032	100
**Hg**	2.26	0.518–5.85	100	2.57	0.421–7.05	100	0.474	0.072–1.20	100	0.544	0.104–1.00	100
**Pb**	8.55	4.68–14.5	100	13.7	5.99–29.9	100	<0.184		0	0.404	<0.184–0.404	7
**Bi**	<0.029		0	<0.029		0	<0.019		0	<0.019		0
**Th**	0.016	<0.010–0.019	29	0.018	<0.010–0.018	7	<0.005		0	<0.005		0
**U**	<0.006		0	<0.006		0	0.006	<0.004–0.006	7	<0.004		0

Concentrations in *µ*g/L; ^*∗*^concentrations in mg/L. With bold text for single elements and italic numbers for detection frequencies.

**Table 11:** Concentrations in selected real samples from nonpregnant and pregnant women from the MISA study (*N* = 15 + 15).

Element	Nonpregnant women			Pregnant women		
	Concentrations		Detected	Concentrations			Detected
	Mean	Range	(%)	Mean	Range		(%)
**Li**	31.2	2.22–280	*100*	7.77	2.37–22.2		*100*
**Be**	<0.512		*0*	<0.512			*0*
**V**	0.431	0.116–0.774	*100*	0.406	0.068–0.871		*100*
**Co**	0.709	0.076–3.22	*100*	0.377	0.085–1.79		*100*
**Ni**	2.39	0.413–12.0	*100*	1.31	0.58–2.96		*100*
**Se**	29.2	7.88–56.8	*100*	34.0	11.8–73.7		*100*
**Sn**	0.510	0.087–1.67	*100*	0.435	<0.063–1.10		*87*
**I**	113	19.3–316	*100*	135	47.7–260		*100*
**Al**	<10.3		*0*	13.2	<10.3–13.2		*7*
**Cr**	0.275	<0.173–0.312	*33*	0.338	<0.173–0.529		*73*
**Mn**	0.243	<0.088–0.453	*80*	0.220	<0.088–0.384		*67*
**Cu**	11.2	4.32–26.5	*100*	8.54	4.12–18.6		*100*
**Zn**	482	96.1–1070	*100*	227	43.6–489		*100*
**As**	70.4	0.801–536	*100*	26.7	1.30–86.7		*100*
**Sr**	141	45.1–445	*100*	136	55.0–221		*100*
**Zr**	0.046	<0.035–0.062	*33*	0.051	<0.035–0.096		*67*
**Mo**	47.2	11.3–144	*100*	48.1	26.4–124		*100*
**Pd**	0.040	<0.027–0.065	*53*	0.040	<0.020–0.074		*40*
**Ag**	0.031	<0.019–0.037	*40*	0.035	<0.019–0.047		*27*
**Cd**	0.360	<0.153–0.615	*87*	0.338	<0.153–0.535		*53*
**Sb**	0.131	<0.110–0.148	*20*	0.188	<0.110–0.298		*53*
**Te**	0.548	<0.548–0.641	*27*		<0.358–0.378		*7*
**Ba**	2.62	0.643–7.37	*100*	1.77	0.162–5.56		*100*
**Ce**	0.011	<0.009–0.011	*7*	<0.009			*0*
**W**	0.101	0.034–0.215	*100*	0.094	0.044–0.248		*100*
**Pt**	0.005	<0.004–0.005	*7*	<0.004			*0*
**Tl**	0.243	0.054–0.444	*100*	0.196	0.057–0.405		*100*
**Hg**	0.908	<0.041–5.19	*47*	0.170	<0.041–0.594		*80*
**Pb**	0.980	<0.325–2.11	*60*	1.54	<0.325–4.84		*33*
**Bi**	0.588	<0.285–0.588	*7*	<0.285			*0*
**Th**	<0.004		*0*	<0.004			*0*
**U**	<0.004		*0*		<0.004–0.015		*7*

Concentrations in *µ*g/L. With bold text for single elements and italic numbers for detection frequencies.

**Table 12:** Clinical reference and action concentrations for selected toxic and trace elements in whole blood and serum.

Matrix	Element	MDL	MQL	Reference concentration		Recommended treatment	Toxic concentration	References
				Lower	Upper			
Whole blood	**Cd**	0.022	0.072	0	0.866		50.0	[65]
	**Hg**	0.004	0.012	0	5.01	>15	100	[65]
	**Pb**	0.097	0.322	2.07	26.9	>441	>0	[65]

Serum	**Li ** ^*∗*^	0.004	0.013				10.5	[65]
	**Co**	0.005	0.017		<0.884	∼5.89		[65]
	**Se**	1.16	3.87	15.8	126		403	[65]
	**Al**	8.38	27.9		<5.4	>60	>200	[65]
	**Cr**	0.543	1.81		<0.302	∼5.20		[65]
	**I ** ^*∗*^	0.0001	0.0002	40	92	>80	>250	[66]
	**Cu ** ^*∗*^	0.015	0.050	0.699	2.54			[65]
	**Zn ** ^*∗*^	0.011	0.037	0.523	1.24			[65]
	**Mo**	0.023	0.076	0.384	2.01	>9.60		[65]

Concentrations in *µ*g/L; ^*∗*^concentrations in mg/L. With bold text for single elements.

## Data Availability

The data for validating the method and demonstrating its applicability used to support the findings of this study are included within the article and the Supplementary Materials.

## Supplementary Materials

Supplementary Materials Supplementary Material file with the title “A fast-forward dilute-and-shoot multielement method for analysis of 33 elements in human whole blood, serum, and urine by inductively coupled plasma mass spectrometry: A streamlined approach for clinical diagnostic and biomonitoring” is available online and contains the following: Table S1: overview on elements of interest with calibration samples applied; Table S2: overview on single standards from Inorganic Ventures; Table S3: ClinCal® calibrators and assigned calibration concentrations for analysis of whole blood, serum, and urine; Table S4: MS parameters; Table S5: method detection limits and method quantification limits; Table S6: measured concentrations for external quality assurance programme whole blood samples (QMEQAS) for elements without assigned concentrations; Table S7: precision and repeatability for analysis of ClinChek (A) and Seronorm (B) whole blood samples for elements without given reference concentrations; Table S8: measured concentrations for external quality assurance programme serum samples (QMEQAS) for elements without assigned concentrations; Table S9: precision and repeatability for analysis of ClinChek serum samples for elements without given reference concentrations; Table S10: measured concentrations for external quality assurance programme urine samples (QMEQAS) for elements without assigned concentrations; Table S11: precision and repeatability for analysis of ClinChek and Seronorm urine samples for elements without given reference concentrations; Figure S1: worktable on the Freedom Evo 200.
