# Supplementary material for: A Fast-Forward Dilute-and-Shoot Multielement Method for Analysis of 33 Elements in Human Whole Blood, Serum, and Urine by Inductively Coupled Plasma Mass Spectrometry: A Streamlined Approach for Clinical Diagnostic and Biomonitoring
Source: J Anal Methods Chem. 2024 Aug 20;2024:9944995. doi: 10.1155/2024/9944995 (PMC11535262; doi:10.1155/2024/9944995)
Supplement: Supplementary Materials — Supplementary Material file with the title “A fast-forward dilute-and-shoot multielement method for analysis of 33 elements in human whole blood, serum, and urine by inductively coupled plasma mass spectrometry: A streamlined approach for clinical diagnostic and biomonitoring” is available online and contains the following: Table S1: overview on elements of interest with calibration samples applied; Table S2: overview on single standards from Inorganic Ventures; Table S3: ClinCal® calibrators and assigned calibration concentrations for analysis of whole blood, serum, and urine; Table S4: MS parameters; Table S5: method detection limits and method quantification limits; Table S6: measured concentrations for external quality assurance programme whole blood samples (QMEQAS) for elements without assigned concentrations; Table S7: precision and repeatability for analysis of ClinChek (A) and Seronorm (B) whole blood samples for elements without given reference concentrations; Table S8: measured concentrations for external quality assurance programme serum samples (QMEQAS) for elements without assigned concentrations; Table S9: precision and repeatability for analysis of ClinChek serum samples for elements without given reference concentrations; Table S10: measured concentrations for external quality assurance programme urine samples (QMEQAS) for elements without assigned concentrations; Table S11: precision and repeatability for analysis of ClinChek and Seronorm urine samples for elements without given reference concentrations; Figure S1: worktable on the Freedom Evo 200. [file 9944995.f1.pdf]

Supplementary Material

A fast forward dilute and shoot multi-element method for analysis of 33 elements in human whole blood, serum, and urine by inductively coupled plasma mass-spectrometry: A streamlined approach for clinical diagnostic and biomonitoring

Sandra Huber*[a], Jörg Michel[b], Maurice Reijnen[c], Maria Averina[a,d], Bjørn Bolann[e,f], Jon Øyvind Odland[g], Solrunn Hansen[h] and Jan Brox[a]

[a] Department of Laboratory Medicine, University Hospital of North Norway, NO-9038 Tromsø, Norway

[b] Perkin Elmer, DE-63110 Rodgau, Germany

[c] Inorganic Solutions, NL-5371CN Ravenstein, The Netherlands

[d] Department of Clinical Medicine, UiT The Arctic University of Norway, NO-9037 Tromsø, Norway

[e] Department of Clinical Science, University of Bergen, NO-5021 Bergen, Norway

[f] Department of Medical Biochemistry and Pharmacology, Haukeland University Hospital, NO-5021 Bergen, Norway

[g] Bioscience and Aquaculture, Nord University, NO-8049 Bodø, Norway

[h] Department of Health and Care Sciences, UiT The Arctic University of Norway, NO-9037 Tromsø, Norway

Content:

Table S1: Overview on elements of interest with calibration samples applied. Page 3

Table S2: Overview on single standards from Inorganic Ventures. Page 4

Table S3: ClinCal calibrators and assigned calibration concentrations for analysis of whole blood, serum, and urine. Page 4

Figure S1: Worktable on the Freedom Evo 200. Page 5

Table S4: MS-parameters. Page 6

Table S5: Method detection limits and method quantification limits. Page 7

Table S6: Measured concentrations for external quality assurance programme whole blood samples (QMEQAS) for elements without assigned concentrations. Page 8

Table S7: Precision and repeatability for analysis of ClinChek (A) and Seronorm (B) whole blood samples for elements without given reference concentrations. Page 9

Table S8: Measured concentrations for external quality assurance programme serum samples (QMEQAS) for elements without assigned concentrations. Page 10

Table S9: Precision and repeatability for analysis of ClinChek serum samples for elements without given reference concentrations. Page 10

Table S10: Measured concentrations for external quality assurance programme urine samples (QMEQAS) for elements without assigned concentrations. Page 11

Table S11: Precision and repeatability for analysis of ClinChek and Seronorm urine samples for elements without given reference concentrations. Page 11

Table S1: Overview on elements of interest with calibration samples applied. Where reference concentrations were not given, concentrations were measured. If the element of interest was below the MDL, it was spiked to the calibration sample.

Element	Whole blood			Serum			Urine		
	ClinCal (LOT 310)		Spiked	ClinCal (LOT 538, 1318)		Spiked	ClinCal (LOT 1047, 1489)		Spiked
	reference conc.	analysed conc.		reference conc.	analysed conc.		reference conc.	analysed conc.	
Li				x				x	
Be			x	x					x
V			x	x			x		
Co	x			x			x		
Ni	x			x			x		
Se	x			x			x		
Sn	x			x			x		
I		x		x			x		
B			x	x	x	x			
Al			x			x	x		x
Cr	x			x			x		
Mn	x			x			x		
Cu	x			x			x		
Zn	x			x			x		
As	x			x			x		
Sr		x		x				x	
Zr			x			x			x
Mo	x			x			x		
Pd	x			x			x		
Ag	x			x			x		
Cd	x			x			x		
Sb			x	x			x		
Te			x			x			x
Ba		x		x			x		
Ce			x			x			x
W			x			x			x
Pt	x			x			x		
Tl	x			x			x		
Hg	x			x			x		
Pb	x					x	x		x
Bi			x	x					x
Th			x			x			x
U			x			x			x

Table S2: Overview on single standards from Inorganic Ventures.

Element	Abbreviation	Name on the vial	Concentration [µg/mL]	Accuracy [µg/mL]	Parts per million [ppm]	Solvent	Purity [%]	Application
Aluminium	Al	MSAL10PPM	10.0	± 0.056	10	3% (v/v) HNO3	99.999	spike mixture
Gold	Au	MATRIKS-104	1.00	± 0.007	1	2% (v/v) HNO3	n.a.	stabiliser diluent
Boron	B	MSB10PPM	10.0	± 0.077	10	0.1% (v/v) HNO3	100.000	spike mixture for whole blood and serum
Beryllium	Be	MSBE-10PPM	10.0	± 0.064	10	3% (v/v) HNO3	100.000	spike mixture for whole blood and urine
Bismuth	Bi	MSBI10PPM	10.0	± 0.063	10	5% (v/v) HNO3	99.999	spike mixture for whole blood and urine
Cerium	Ce	MSCE10PPM	10.0	± 0.060	10	7% (v/v) HNO3	99.998	spike mixture
Lead	Pb	MSPB10PPM	10.0	± 0.056	10	0.5% (v/v) HNO3	99.999	spike mixture for serum and urine
Rhenium	Re	CGRE1	1004	± 2	1000	3% (v/v) HNO3	99.998	internal standard
Rhodium	Rh	CGRH1	999	± 5	1000	15% (v/v) HCl	99.998	internal standard
Antimony	Sb	MSSB10PPM	9.99	± 0.085	10	1% (v/v) HNO3	99.995	spike mixture for whole blood and urine
Tellurium	Te	MSTEN10PPM	10.0	± 0.066	10	10% (v/v) HNO3	99.986	spike mixture
Thorium	Th	MSTH10PPM	10.0	± 0.048	10	4.3% (v/v) HNO3	99.993	spike mixture
Uranium	U	MSU10PPM	10.0	± 0.077	10	1.4% (v/v) HNO3	100.000	spike mixture
Vanadium	V	MSV10PPM	10.0	± 0.062	10	2% (v/v) HNO3	99.999	spike mixture for whole blood and urine
Tungsten	W	MSW10PPM	10.0	± 0.076	10	0.14% (v/v) HNO3	99.995	spike mixture
Zirconium	Zr	CGZR1	999	± 5	1000	tr. HF	99.991	ca 1:20 dilution for spike mixture

Table S3: ClinCal calibrators and assigned calibration concentrations for analysis of whole blood, serum, and urine. * indicates mg/L unit for whole blood and serum samples.

		Whole Blood ClinCal LOT 310			Serum ClinCal LOT 358			Urine ClinCal LOT 1047		
Element	unit	standard 1	standard 2	standard 3	standard 1	standard 2	standard 3	standard 1	standard 2	standard 3
Li	µg/L				0.087	0.218	0.437	0.032	0.081	0.161
Be	µg/L	0.39	1.75	14.9	0.187	0.468	0.935	0.493	1.63	12.335
V	µg/L	0.36	1.62	13.8	0.117	0.293	0.585	0.618	1.55	3.09
Co	µg/L	0.31	0.775	1.16	0.072	0.180	0.361	0.393	0.983	1.97
Ni	µg/L	0.298	0.745	1.12	0.075	0.187	0.373	0.384	0.960	1.92
Se	µg/L	3.74	9.35	14.0	1.37	3.43	6.85	1.04	2.60	5.20
Sn	µg/L	0.200	0.500	0.750	0.274	0.685	1.37	0.125	0.313	0.625
I	µg/L	0.800	2.00	3.00	1.04	2.60	5.20	5.96	14.9	29.8
B	µg/L	0.450	2.01	17.1	0.670	2.31	17.5			
Al	µg/L	0.290	1.32	11.2	2.63	8.31	51.6	3.24	9.69	56.2
Cr	µg/L	0.258	0.645	0.969	0.076	0.190	0.379	0.249	0.623	1.245
Mn	µg/L	0.494	1.24	1.85	0.100	0.250	0.500	0.248	0.620	1.24
Cu	µg/L*	0.039	0.098	0.147	0.017	0.043	0.087	1.40	3.50	7.00
Zn	µg/L*	0.169	0.423	0.635	0.018	0.045	0.089	6.22	15.55	31.1
As	µg/L	0.482	1.21	1.81	0.287	0.718	1.44	1.26	3.15	6.3
Sr	µg/L	0.390	1.02	1.52	0.266	0.667	1.24	0.204	0.509	1.02
Zr	µg/L	0.300	1.34	11.4	0.500	1.72	13.1	0.650	2.14	16.3
Mo	µg/L	0.016	0.031	0.051	0.076	0.191	0.382	1.20	3.00	6.00
Pd	µg/L	0.094	0.235	0.351	0.310	0.775	1.55	0.134	0.335	0.670
Ag	µg/L	0.200	0.500	0.750	0.299	0.748	1.50	0.097	0.241	0.483
Cd	µg/L	0.174	0.435	0.653	0.076	0.190	0.379	0.202	0.505	1.01
Sb	µg/L	0.310	0.980	4.82	0.110	0.275	0.550	0.577	1.44	2.89
Te	µg/L	0.110	0.480	4.08	0.180	0.62	4.74	0.220	0.740	5.63
Ba	µg/L	0.029	0.059	0.090	1.94	4.85	9.70	0.773	1.93	3.87
Ce	µg/L	0.090	0.410	3.47	0.170	0.570	4.35	0.200	0.650	4.94
W	µg/L	0.110	0.490	4.18	0.170	0.590	4.45	0.150	0.490	3.71
Pt	µg/L	0.129	0.322	0.483	0.013	0.032	0.065	0.019	0.047	0.094
Tl	µg/L	0.258	0.645	0.968	0.091	0.227	0.453	0.228	0.570	1.14
Hg	µg/L	0.169	0.423	0.634	0.112	0.280	0.560	0.324	0.810	1.62
Pb	µg/L	7.14	17.9	26.8	0.270	0.950	7.18	1.04	2.85	11.7
Bi	µg/L	0.620	2.80	23.8	0.071	0.178	0.355	0.930	3.08	23.5
Th	µg/L	0.110	0.480	4.12	0.150	0.530	4.05	0.100	0.340	2.59
U	µg/L	0.100	0.440	3.77	0.120	0.410	3.11	0.100	0.320	2.41

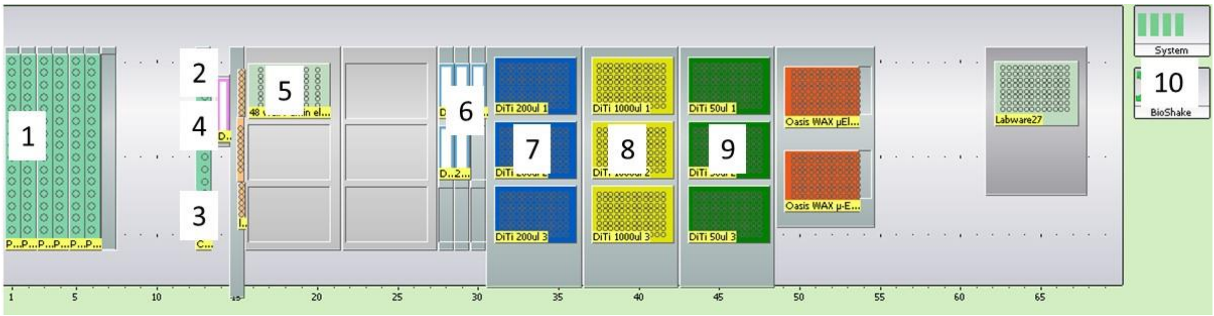

Figure S1: Worktable on the Freedom Evo 200. **1** samples (40), **2** calibration sample (1), **3** blank samples (5), **4** spike mixtures (3), **5** Perkin Elmer 7.5 mL 48-well plate, **6** reagents, **7** 200 µL disposable tips, **8** 1 mL disposable tips, **9** 50 µL disposable tips, **10** shaker.

Table S4: MS parameters with isotopes, dwell times, integration times, corrections, and KED gas flows applied for analysis in whole blood, serum, and urine samples. Parameters given in parenthesis are valid for urine. * indicates internal standards; ** indicates stabiliser.

Functions	Analyte	Mass [amu]	Dwell time per amu [ms]	Integration time [ms]	KED gas flow [mL]	Corrections	Potential Interferences
	Li	7.0					
	Be	9.0	100	2000	4.8		
	V	50.9	50	1000	4.8		ClO, HSO
	Co	58.9	50	1000	4.8		CaO
1	Ni	59.9 / 61.9	100	2000	4.8		CaO / TiO
	Se	77.9 / 81.9	100	2000	4.8	- 0.030461 * Kr 83 / - 1.007833 * Kr 83	Kr, Ar2, Gd++, Gd++, Dy++ / Kr, BrH, Ar2H, Ho++, Dy++, Er++
	Sn	117.9	50	1000	4.8		MoO, U++
	I	126.9	50	1000	4.8		MoO2
	Rh-1*	102.9	50	1000	4.8		
	B	11.0	50	1000	5.7		BH
	Al	27.0	100	2000	5.7		BO, CN, BeO
	Cr	51.9	100	2000	5.7		ArN, ClO, ArO, SO, ArC, HClO
	Mn	54.9	100	2000	5.7		ArN, HClO, ClO
	Cu	62.9	50	1000	5.7		PO2, TiO
	Zn	65.9	50	1000	5.7		TiO, VO, SO2, Ba++
	As	74.9	100	2000	5.7		ArCl, Sm++, Nd++, Eu++
	Sr	87.9	50	1000	5.7		Yb++, Lu++
	Zr	89.9	100	2000	5.7		
	Mo	97.9	50	1000	5.7	- 0.109613 * Ru 101	Ru, BrO
2	Rh-2*	102.9	50	1000	5.7		
	Pd	105.9	50	1000	5.7	- 0.097656 * Cd 111	Cd, SrO, ZrO, YO
	Ag	106.9	50	1000	5.7		YO, ZrO
	Cd	110.9	50	1000	5.7		MoO
	Sb	120.9	50	1000	5.7		
	Te	129.9	100	2000	5.7	- 0.009437 * Ba 137 - 0.154312 * Xe 129	Ba, Xe, MoO2
	Ba	137.9	50	1000	5.7	- 0.000901 * La 139 - 0.002838 * Ce 140	La, Ce
	Ce	139.9	100	2000	5.7		
	W	184.0	50	1000	5.7	- 0.001238 * Os 189	Os, ErO, YbO
	Re*	187.0	50	1000	5.7	- 0.121362 * Os 189	Os, YbO, TmO
	Pt	195.0	50	1000	5.7		HfO
	Au**	197.0	50	1000	5.7		TaO, HfO
3	Tl	205.0	50	1000	5.7		
	Hg	200.0 / 202.0	100 (50)	2000 (1000)	5.7		WO
	Pb	207.0+208.0 +209.0	50	1000	5.7		
	Bi	209.0	50	1000	5.7		
	Th	232.0	100	2000	5.7		
	U	238.1	50	1000	5.7		

Table S5: Method detection limits and method quantification limits for toxic and essential elements in human whole blood, serum, and urine based on blank samples (whole blood N = 21, serum N = 33, urine N = 49). Concentrations in µg/L; * concentrations in mg/L.

Element	Whole blood		Serum		Urine	
	MDLs	MQLs	MDLs	MQLs	MDLs	MQLs
Li*			0.004	0.013	0.071	0.236
Be	0.141	0.469	0.109	0.362	0.512	1.71
V	0.217	0.723	0.224	0.745	0.039	0.131
Co	0.001	0.003	0.005	0.017	0.044	0.148
Ni	0.057	0.188	0.176	0.587	0.178	0.594
Se	0.503	1.68	1.16	3.87	1.39	4.65
Sn	0.112	0.373	0.035	0.117	0.063	0.211
I	0.193	0.643	0.057	0.191	0.175	0.584
B	3.91	13.0				
Al	7.08	23.6	8.38	27.9	10.3	34.3
Cr	0.163	0.544	0.543	1.81	0.173	0.577
Mn	0.060	0.201	0.201	0.671	0.088	0.293
Cu*	0.048	0.161	0.015	0.050	3.73	12.4
Zn*	0.001	0.004	0.011	0.037	3.26	10.9
As	0.047	0.156	0.052	0.173	0.114	0.380
Sr	0.105	0.351	0.169	0.564	0.179	0.598
Zr	0.027	0.090	0.075	0.251	0.035	0.115
Mo	0.030	0.100	0.023	0.076	0.027	0.091
Pd	0.030	0.100	0.003	0.011	0.020	0.067
Ag	0.009	0.030	0.054	0.182	0.019	0.065
Cd	0.022	0.072	0.062	0.206	0.153	0.509
Sb	0.035	0.118	0.079	0.263	0.110	0.365
Te	0.337	1.12	0.365	1.22	0.358	1.19
Ba	0.224	0.748	0.528	1.76	0.095	0.316
Ce	0.010	0.033	0.009	0.030	0.009	0.030
W	0.017	0.058	0.005	0.016	0.004	0.012
Pt	0.003	0.012	0.018	0.061	0.004	0.013
Tl	0.027	0.089	0.004	0.012	0.004	0.012
Hg	0.004	0.012	0.020	0.065	0.041	0.137
Pb	0.097	0.322	0.184	0.615	0.325	1.08
Bi	0.029	0.097	0.019	0.064	0.285	0.951
Th	0.010	0.033	0.005	0.017	0.004	0.012
U	0.006	0.021	0.001	0.005	0.010	0.032

Table S6: Measured concentrations for external quality assurance programme whole blood samples (QMEQAS) for elements without assigned concentrations together with variation coefficients of three prepared and measured aliquots. Concentrations in µg/L; * concentrations in mg/L.

Element	QM-B-Q1911		QM-B-1921		QM-B-Q2001		QM-B-Q2012	
	measured concentrations	**CV [%]**	***measured concentrations***	**CV [%]**	***measured concentrations***	**CV [%]**	**measured.** ***concentrations***	**CV [%]**
**B**	< 3.91		28.4	**2.9**	46.7	**13**	18.9	**35**
**Zr**	< 0.027		0.052	**20**	< 0.027		< 0.027	
**Pd**	< 0.030		< 0.030		< 0.030		< 0.030	
**Ce**	< 0.010		0.019	**9.9**	0.018	**30**	0.026	**33**
**W**	< 0.017		< 0.017		< 0.017		< 0.017	

Table S7: Precision and repeatability for analysis of ClinChek (A) and Seronorm (B) whole blood samples for elements without given reference. Measured concentration for control materials (N= 3 × 6). Intra-day variation coefficients (CVs) (N = 6) and inter-day CVs (N = 6 + 6 + 6 + 11). Method detection limits based on blank samples (N=21). Concentrations in µg/L.

**A**		**ClinChek L1 (Lot 445)**						**ClinChek L2 (Lot 455)**						**ClinChek L3 (Lot 445)**					
**Element**	**MDLs**	**concentrations**			**deviation**	**CVs [%]**		**concentrations**			**deviation**	**CVs [%]**		**concentrations**			**deviation**	**CVs [%]**	
		**target**	**range**	**measured**	**[%]**	intra-day	**inter-day**	**target**	**range**	**measured**	**[%]**	intra-day	**inter-day**	**target**	**range**	**measured**	**[%]**	intra-day	**inter-day**
**Be**	0.141			< 0.141						< 0.141						< 0.141			
**V**	0.217			< 0.217						< 0.217						< 0.217			
**I**	0.193			37.1		3.0	**5.9**			36.9		3.2	**6.4**			36.8		1.9	**7.0**
**B**	3.91			23.0		29	**30**			25.2		19	**35**			22.3		43	**39**
**Al**	7.08			< 7.08						< 7.08						< 7.08			
**Sr**	0.105			20.5		2.1	**7.0**			20.3		3.8	**7.9**			20.9		2.5	**7.1**
**Zr**	0.027			< 0.027						< 0.027						< 0.027			
**Pd**	0.030			0.936		8.5	**7.9**			1.71		6.2	**7.2**			4.04		2.4	**5.8**
**Te**	0.337			< 0.337						< 0.337						< 0.337			
**Ba**	0.224			0.694		12	**22**			0.673		14	**19**			0.714		5.8	**22**
**Ce**	0.010			< 0.010						< 0.010						< 0.010			
**W**	0.017			< 0.017						< 0.017						< 0.017			
**Bi**	0.029			< 0.029						< 0.029						< 0.029			
**Th**	0.010			< 0.010						< 0.010						< 0.010			
**U**	0.006			< 0.006						< 0.006						< 0.006			

**B**		**Sero L1 (Lot 1406263)**						**Sero L2 (Lot 1406264)**						**Sero L3 (Lot 1509408)**					
**Element**	**MDLs**	**concentrations**			**deviation**	**CVs [%]**		**concentrations**			**deviation**	**CVs [%]**		**concentrations**			**deviation**	**CVs [%]**	
		**target**	**range**	**measured**	**[%]**	intra-day	**inter-day**	**target**	**range**	**measured**	**[%]**	intra-day	**inter-day**	**target**	**range**	**measured**	**[%]**	intra-day	**inter-day**
**Pd**	0.030			< 0.030						< 0.030						< 0.030			

Table S8: Measured concentrations for external quality assurance programme serum samples (QMEQAS) for elements without assigned concentrations together with variation coefficients of three prepared and measured aliquots. Concentrations in µg/L; * concentrations in mg/L.

Element	QM-S-Q1925		QM-S-Q2025		QM-S-Q2116		QM-S-Q2117	
	measured concentrations	CV [%]	measured concentrations	CV [%]	measured concentrations	CV [%]	measured concentrations	CV [%]
**Li***	< 0.004		< 0.004		< 0.004		< 0.004	
**Sr**	27.9	**4.1**	23.5	**4.1**	23.8	**1.3**	28.9	**3.5**
**Zr**	< 0.075		< 0.075		< 0.075		< 0.075	
**Pd**	< 0.003		< 0.003		< 0.003		< 0.003	
**Ce**	0.015	**41**	0.015	**16**	< 0.009		0.015	**22**
**W**	< 0.005		< 0.005		< 0.005		< 0.005	

Table S9: Precision and repeatability for analysis of ClinChek serum samples for elements without given reference concentrations. Measured concentration for control materials (N= 3 × 8). Intra-day variation coefficients (CVs) (N = 8) and inter-day CVs (N = 8 + 8 + 8). Method detection limits based on blank samples (N=33). Concentrations in µg/L.

Element	MDLs	ClinChek L1 (Lot 544)			ClinChek L1 (Lot 544)		
			CVs [%]			CVs [%]	
		**measured**	intra-day	**inter-day**	**measured**	intra-day	**inter-day**
**B**	1.39	< 1.39			161	7.3	**11**
**Mn**	0.201	2.04	12	**10**			
**Sr**	0.169	41.9	3.0	**6.8**	24.3	3.3	**8.3**
**Zr**	0.075	0.391	9.2	**16**	0.151	16	**13**
**Te**	0.365	< 0.365			< 0.365		
**Ce**	0.009	0.021	43	**70**	0.534	2.4	**8.5**
**W**	0.005	0.979	2.9	**2.7**	0.034	11	**21**
**Pb**	0.184	1.27	2.8	**11**	0.278	14	**58**
**Bi**	0.019	0.845	2.9	**3.6**			
**Th**	0.010	< 0.010			< 0.010		
**U**	0.001	0.070	5.3	**12**	0.015	11	**18**

Table S10: Measured concentrations for external quality assurance programme urine samples (QMEQAS) for elements without assigned concentrations together with variation coefficients of three prepared and measured aliquots. Concentrations in µg/L.

Element	QM-U-Q2106		QM-U-Q2114		QM-U-Q2123		QM-U-Q2124	
	measured concentration	CV [%]	measured concentration	CV [%]	measured concentration	CV [%]	measured concentration	CV [%]
**Zr**	0.049	**20**	0.043	**30**	< 0.035		0.043	**1.7**
**Pd**	< 0.020		0.023	**44**	< 0.020		< 0.020	
**Ce**	< 0.009		< 0.009		< 0.009		0.007	**8.4**
**W**	0.157	**11**	1.62	**0.9**	0.220	**6.9**	0.070	**13**

Table S11: Precision and repeatability for analysis of ClinChek and Seronorm urine samples for elements without given reference concentrations. Measured concentration for control materials (N= 3 × 8). Intra-day variation coefficients (CVs) (N = 8); inter-day CVs (N = 8 + 8 + 8). Method detection limits based on blank samples (N=49). Concentrations in µg/L.

Element	MDLs	ClinChek L1 (Lot 1227)			ClinChek L2 (Lot 1227)			Sero L1 (1706877)			Sero L2 (1403081)		
			CVs [%]			CVs [%]			CVs [%]			CVs [%]	
		measured	intra-day	inter-day	measured	intra-day	inter-day	measured	intra-day	inter-day	measured	intra-day	inter-day
**Li**	0.071	6.41	3.0	**6.0**	5.64	3.1	**5.3**						
**Sr**	0.179	42.5	3.0	**5.5**	42.3	2.7	**4.9**						
**Zr**	0.035	< 0.035			< 0.035			< 0.035					
**Pd**	0.020							<0.020			<0.020		
**Ag**	0.019	1.32	5.1	**12**	4.37	2.3	**11**						
**Te**	0.358	< 0.358			< 0.358								
**Ce**	0.009	< 0.009			< 0.009								
**W**	0.004	0.024	18	**17**	0.027	9.6	**19**	0.031	19	**17**	0.159	4.3	**9.4**
**Bi**	0.285	< 0.285			< 0.285								
**Th**	0.004	< 0.004			< 0.004								
**U**	0.010	< 0.010			< 0.010								
